# Calophyllaceae plastomes, their structure and insights in relationships within the clusioids

**DOI:** 10.1038/s41598-021-99178-z

**Published:** 2021-10-20

**Authors:** Rafaela Jorge Trad, Fernanda Nunes Cabral, Volker Bittrich, Saura Rodrigues da Silva, Maria do Carmo Estanislau do Amaral

**Affiliations:** 1 Department of Plant Biology, Biology Institute, University of Campinas (UNICAMP), CP 6109, Campinas, SP 13083-970 Brazil; 2 Macroecology Lab @ J3-166, Institute of Biological Sciences – ICB, Federal University of Minas Gerais (UFMG), Belo Horizonte, Campinas, MG 31270-901 Brazil; 3 Departamento de Ciências e Linguagens, Instituto Federal de Minas Gerais – Campus Bambuí, Bambuí, MG 38900-000 Brazil; 4 Department of Technology, UNESP - São Paulo State University, Campus Jaboticabal, Jaboticabal, SP 14884-900 Brazil; 5 Volker Bittrich is an independent scientist, Campinas, Brazil

**Keywords:** Phylogenetics, Taxonomy, Plant evolution

## Abstract

A complete chloroplast genome is not yet available for numerous species of plants. Among the groups that lack plastome information is the clusioid clade (Malpighiales), which includes five families: Bonnetiaceae, Calophyllaceae, Clusiaceae, Hypericaceae, and Podostemaceae. With around 2200 species, it has few published plastomes and most of them are from Podostemaceae. Here we assembled and compared six plastomes from members of the clusioids: five from Calophyllaceae (newly sequenced) and one from Clusiaceae. Putative regions for evolutionary studies were identified and the newly assembled chloroplasts were analyzed with other available chloroplasts for the group, focusing on Calophyllaceae. Our results mostly agree with recent studies which found a general conserved structure, except for the two Podostemaceae species that have a large inversion (*trn*K-UUU–*rbc*L) and lack one intron from *ycf*3. Within Calophyllaceae we observed a longer LSC and reduced IRs in *Mahurea exstipulata*, resulting in some genic rearrangement, and a short inversion (*psb*J–*psb*E) in *Kielmeyera coriacea*. Phylogenetic analyses recovered the clusioids and the five families as monophyletic and revealed that conflicts in relationships reported in the literature for the group agree with nodes concentrating uninformative or conflicting gene trees. Our study brings new insights about clusioid plastome architecture and its evolution.

## Introduction

Since the advance of next-generation sequencing complete plastomes or chloroplast coding sequences have been the most used source of information to explore phylogenetic relationships among plants at diverse scales^1–6^. This is due to their high number of copies in a cell (1000 to 10,000^7^), their much smaller size when compared to nuclear genomes (ca. 120 genes^8^ *vs.* thousands of genes^9^), and the availability of numerous tools to assemble chloroplast genomes (e.g., NOVOPlasty^10^, GetOrganelle^11^, Geneious^12^, MITOBim^13^). Thus, it usually is easier to obtain the DNA from the chloroplast when compared to the nucleus. In addition, sequencing the chloroplast is cost advantageous, and the generated data require less computational power to be processed and analyzed. Despite the existence of a canonical chloroplast structure for land plants^14^, which includes two unique regions (the large single copy-LSC, and the small single copy-SSC), and one duplicated region (the inverted repeat-IR), much variation has been already found at different taxonomic levels^1–6^. Finally, most of the chloroplast genes are functional, thus usually conserved^15^. These reasons make plastid DNA extremely attractive for high throughput sequencing. Furthermore, the possibility of using the chloroplast genome from a phylogenetically distant taxon as a reference for the assembly accelerates data processing. However, it is important to mention that the analysis of nuclear genes is becoming much easier^16^.

Although the chloroplast genome is generally imagined as being a quadripartite circular structure^7,8,17^, there is evidence that these circular molecules represent only a small fraction of the total chloroplast DNA^18^. Indeed, chloroplast chromosomes are hypothesized to consist of branched and complex multigenomic forms^18,19^. Even though phylogenetic conflict within plastid data has been known for over two decades^20,21^, it was largely neglected until recently, when the traditional approach of treating the plastome as a single “supergene” has been questioned^22,23^, creating some debate (see Doyle 2021)^24^. There is a growing body of evidence showing that plastid regions contribute differentially to phylogenetic support^22,25–27^ and that several of the plastome genes actually are uninformative^25,26^. Finally, heteroplasmy was recently quantified and may be more common than previously expected^28^. This phenomenon allows the occurrence of heteroplasmic recombination, which is a potential source of gene tree conflict^25^. Besides heteroplasmic recombination, heterotachy, stochastic and systematic errors and horizontal gene transfer have been invoked as potential causes of gene tree conflict. These findings are already changing how we see plastomes and will probably influence future works using plastid data.

The highly diverse order Malpighiales includes 36 recognized families in its most recent circumscription and represents ca. 7.8% of eudicot diversity^29–32^. Intrafamilial relationships remained poorly understood until Xi and collaborators (2012)^33^ presented a phylogeny based on 82 plastid, six mitochondrial, and three nuclear genes and suggested relationships between families in the order, albeit some were only poorly supported. Indeed, recently Cai and collaborators (2021)^34^ described what they called “the perfect storm”—a combination of incomplete lineage sorting, introgression, and gene tree estimation error that may occur simultaneously during periods of rapid radiation, making phylogenetic inference challenging—and showed the difficulties in resolving relationships especially along the backbone of Malpighiales. Within the order, the clusioid clade includes five families (Bonnetiaceae, Calophyllaceae, Clusiaceae, Hypericaceae and Podostemaceae), 94 genera and ca. 2200 species^31,33,35,36^. Relationships within the clusioids had remained unclear—indeed, the very existence of the group was questionable—until somewhat over a decade ago. For example, Wawra (1886)^37^ included some of the taxa in Ternstroemiaceae, today included in the Theaceae (Ericales), and Cronquist (1981)^38^ separated them in different orders and subclasses, while where Podostemaceae were to be placed was almost literally anybody’s guess. On the other hand, the families Hypericaceae and Calophyllaceae have frequently been included in Clusiaceae *s.l.* (sometimes called Guttiferae).

The relationships between the families included in the clusioid clade were explored in more depth by Ruhfel and collaborators (2011, 2013)^35,36^. The authors recognized two major clades: (Clusiaceae + Bonnetiaceae) and (Calophyllaceae (Hypericaceae + Podostemaceae)). Xi and collaborators (2012)^33^ recovered the five clusioid families as monophyletic and in a well-supported clade and the same topology from Ruhfel et al. (2011, 2013)^35,36^; the clade Bonnetiaceae + Clusiaceae had the lowest support (85% bootstrap/0.92 Bayesian posterior probability). Recently, Cai and collaborators (2021)^34^ also recovered the clusioid families in a well-supported clade using 423 single-copy nuclear loci, but internal relationships were very different—(Hypericaceae (Clusiaceae (Calophyllaceae + Bonnetiaceae)))—although poorly supported; Podostemaceae were not included. The conflicting relationships within the clusioids may result from the above-mentioned factors, i.e., “the perfect storm”, but also indicate that nuclear and plastid data have conflicting evolutionary stories, since most of Xi et al.^33^ data came from plastid genes. The use of chloroplast genomic data to investigate intergeneric relationships and/or genome structure within the clusioids is recent. The studies have focused mainly on Clusiaceae^6^ and Podostemaceae^39^. Jin and collaborators (2020)^40^ included Calophyllaceae, but only one plastome, i.e., that of *Mesua ferrea* L.

The family Calophyllaceae is pantropical and includes 14 genera and ca. 460 species^31^. Within the Calophyllaceae two tribes are currently recognized: Endodesmiae, including two monotypic African genera (*Endodesmia* Benth. and *Lebrunia* Staner), and Calophylleae, including the remaining 12 genera (*Calophyllum* L., *Caraipa* Aubl., *Clusiella* Planch. & Triana, *Haploclathra* Benth., *Kayea* Wall., *Kielmeyera* Mart. & Zucc., *Mammea* L., *Mahurea* Aubl., *Marila* Sw., *Mesua* L., *Neotatea* Maguire, *Poeciloneuron* Bedd.), which are distributed throughout the tropics. In both Ruhfel et al*.*^35,41^ and Cabral et al*.*^42^ *Endodesmia* was well-supported as sister to the remaining genera of the family (*Lebrunia* was not included in either study). Relationships among the genera in Calophylleae are not completely understood, e.g., the position of *Calophyllum* + *Mesua* clade may be (1) sister to the clade *Kayea* + *Poeciloneuron*^43^, (2) sister to a clade that includes *Mammea*, *Kayea* and *Poeciloneuron*^35^, or (3) sister to a clade including the neotropical genera *Caraipa*, *Clusiella*, *Haploclathra*, *Kielmeyera*, *Mahurea*, and *Marila*^42^.

Complete plastome information, including its annotation, is still not available for many species. Thus, the present work aims to reduce this knowledge gap for the family Calophyllaceae by presenting four complete and one nearly complete newly assembled and annotated plastid genomes from four different genera in the family (*Calophyllum*, *Caraipa*, *Kielmeyera* and *Mahurea*), represented by the species *Calophyllum brasiliense* Cambess., *Caraipa heterocarpa* Ducke, *Kielmeyera appariciana* Saddi, *K. coriacea* Mart. & Zucc., and *Mahurea exstipulata* Benth. We also assembled and annotated the first plastid genome from *Clusia* L., the largest genus in the Clusiaceae. Our data were analyzed in the context of the clusioid clade, and we hope to improve our understanding of the evolution of the group.

## Results

### Chloroplast structure

**Figure 1:**
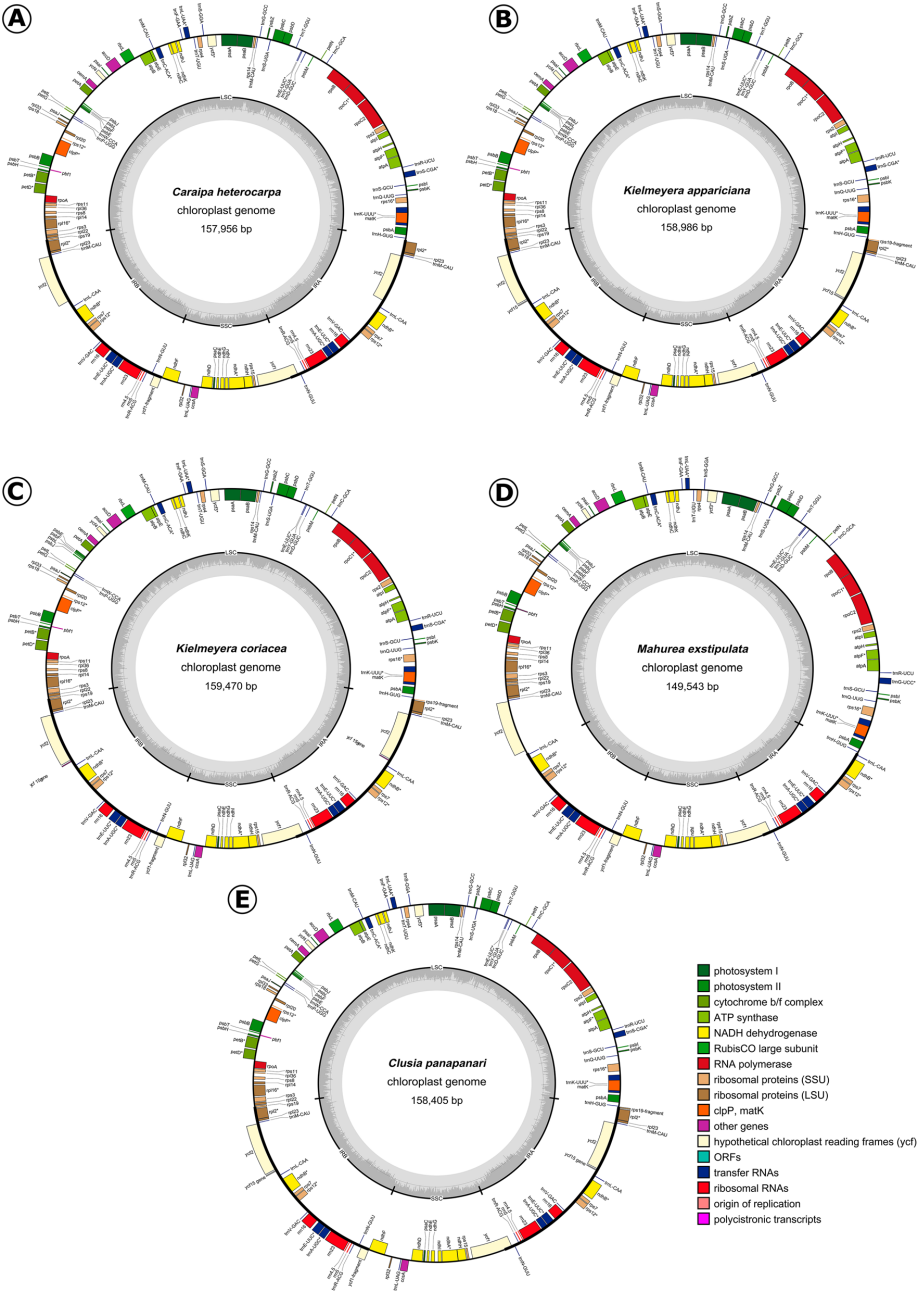
Circular map of the five complete clusioid plastomes. (**A**) *Caraipa heterocarpa*, (**B**) *Kielmeyera appariciana*, (**C**) *K. coriacea*, (**D**) *Mahurea exstipulata*, (**E**) *Clusia panapanari*. The genes represented outside the circle are transcribed counterclockwise and those inside the outer circle are transcribed clockwise. Genes are colored according to their functional groups following the legend. The inner gray graphs indicate the GC content across the plastome.

The newly assembled plastomes presented a quadripartite structure with one LSC, one SSC and two IRs (Fig. 1). The total length ranged from 149,535 bp in *Mahurea exstipulata* to 160,253 bp in *Calophyllum brasiliense*, with mean depth coverage ranging from 52.0 to 474.7 reads. LSC, SSC and IR sizes and their respective GC content are presented in Table 1. Among the newly assembled plastomes *M. exstipulata* showed the most distinct plastome with the largest LSC (98,042 bp) and the smallest IRs (16,553 bp), respectively 9,923 to 12,143 bp longer and 8,966 to 10,781 bp shorter respectively than those of the other species. All species presented an SSC similar in size ranging from 17,464 bp in *C. brasiliense* to 19,102 bp in *Clusia panapanari* (Aubl.) Choisy. The GC content was similar among all species (Table 1).

**Table 1:** GenBank accession numbers and comparison of chloroplast genome size and GC content across three different regions (LSC, SSC, and IR) for 12 clusioid species. *LSC* large single copy, *SSC* small single copy, *IR* inverted repeat.

Species	Family	GenBank	Coverage (mean)	Total reads	Mapped reads	LSC		SSC		IR		Full plastome	
						bp	GC %	bp	GC %	bp	GC %	bp	GC %
*Bonnetia paniculata* Spruce ex. Benth.	Bonnetiaceae	MK995182	–	–	–	84,024	33.9	18,140	29.9	27,309	41.9	156,782	36.2
*Calophyllum brasiliense* Cambess.	Calophyllaceae	MW853786	52.0	8,922,582	61,669	88,119	34.2	17,464	30.6	27,334	42.2	160,253	36.5
*Caraipa heterocarpa* Ducke	Calophyllaceae	MW853787	44.1	8,486,444	49,148	86,990	34.2	18,260	30.7	26,353	42.7	157,956	36.6
*Kielmeyera appariciana* Saddi	Calophyllaceae	MW853788	447.3	8,424,602	504,031	87,648	34.2	18,300	30.5	25,519	42.7	158,986	36.6
*Kielmeyera coriacea* Mart. & Zucc.	Calophyllaceae	MW853789	474.7	11,077,404	540,562	88,263	34.3	18,219	30.6	26,490	42.7	159,470	36.6
*Mahurea exstipulata* Benth.	Calophyllaceae	MW853790	298.2	7,486,882	333,152	98,042	34.6	18,395	30.7	16,553	45.4	149,535	36.5
*Mesua ferrea* L.	Calophyllaceae	MK995181	–	–	–	88,784	34.0	17,482	30.6	27,614	42.1	161,494	36.4
*Clusia panapanari* (Aubl.) Choisy	Clusiaceae	SRR7518735	78.6	2,452,164	89,207	85,899	33.6	19,102	29.5	26,702	42.3	158,405	36.0
*Cratoxylum cochinchinense* (Lour.) Blume	Clusiaceae	MK995180	–	–	–	85,640	34.0	18,892	29.9	26,272	42.1	157,076	36.2
*Garcinia gummi-gutta* (L.) N.Robson	Clusiaceae	NC_047250	–	–	–	84,998	33.5	17,088	30.3	27,058	42.1	156,202	36.2
*Marathrum foeniculaceum* Bonpl.	Podostemaceae	MK995178	–	–	–	79,506	32.2	12,262	28.0	19,916	43.0	131,600	35.1
*Tristicha trifaria* (Bory ex Willd.) Speng.	Podostemaceae	MK995179	–	–	–	79,002	33.7	12,717	30.7	19,623	43.5	130,967	36.3

Five plastomes have 87 protein-coding genes, 37 transfer RNA (tRNA) and eight ribosomal (rRNA), totaling 132 genes. However, *M. exstipulata* had lost two tRNA genes and one of the copies of the *rpl*2, *rpl*23 and *ycf*2 genes and both copies of the *ycf*15 gene, thus having a total of 125 genes in its plastome. A list of all genes is found in Table 2. Only *clp*P gene has two introns. The clusioids have lost one of the two *ycf*3 introns, thus this gene has two exons and one intron as in Jin et al*.*^40^. Among the duplicated genes in the IRa, there are seven tRNAs, eight CDS (seven in *C. heterocarpa* in which the *ycf*15 is pseudogenized, and four in *M. exstipulata* due to its shorter IR), and four rRNAs (Fig. 1). Overall, the gene content is similar within the clusioids, the two Podostemaceae (*Marathrum foeniculaceum* Bonpl. and *Tristicha trifaria* (Bory ex Willd.) Spreng.) being the most distinct.

**Table 2:** List of genes annotated in the six assembled plastomes: *Calophyllum brasiliense*, *Caraipa heterocarpa*: *Clusia panapanari*, *Kielmeyera appariciana*, *K. coriacea*, and *Mahurea exstipulata*.

Functional annotation	Name of the gene
Photosystem I	psaA, psaB, psaC, psaI, psaJ
Protosystem II	psbA, psbB, psbC, psbD, psbE, psbF, psbH, psbI, psbJ, psbK, psbL, psbM, psbN, psbT, psbZ
Cytochrome b/f complex	petA, petB^i^, petD^i^, petG, petL, petN
ATP synthase	atpA, atpB, atpE, atpF^i^, atpH, atpI
NADH dehydrogenase	ndhA^i^, ndhB^i^ (× 2), ndhC, ndhD, ndhE, ndhF, ndhG, ndhH, ndhI, ndhJ, ndhK, ndhL
RubisCO large subunit	rbcL
RNA polymerase	rpoA, rpoB, rpoC1^i^, rpoC2
Ribosomal proteins (SSU)	rps2, rps3, rps4, rps7 (× 2), rps8, rps11, rps12^iT^, rps14, rps15, rps16^i^, rps18, rps19
Ribosomal proteins (LSU)	rpl2^i^ (2x), rpl14, rpl16^i^, rpl20, rpl22, rpl23 (2x), rpl32, rpl33, rpl36
Other genes	ccsA, clpP^i^, matK, accD, cemA, infA
Transfer RNAs	trnA-UGC^i^ (× 2), trnC-ACA^i^, trnC-GCA, trnD-GUC, trnE-UUC^i^ (× 3), trnF-GAA, trnG-GCC, trnG-UCC^i^*, trnH-GUG, trnK-UUU^i^, trnL-CAA (2x), trnL-UAA^i^**, trnL-UAG, trnM-CAU (× 4)***, trnN-GUU (× 2), trnP-UGG, trnQ-UUG, trnR-ACG (× 2), trnR-UCU, trnS-AGA^i^***, trnS-CGA^i^, trnS-GCU, trnS-GGA, trnS-UGA, trnT-GGU, trnT-UGU, trnV-GAC (× 2), trnW-CCA, trnY-GUA
Ribosomal RNAs	rrn4.5 (× 2), rrn5 (× 2), rrn16 (× 2), rrn23 (× 2)
Hypothetical chloroplast reading frames	ycf1 (2x), ycf2 (2x), ycf3^i^, ycf4, ycf15 (2x)

^i^Genes with introns.

^T^Transpliced gene.

*Present only in *M. exstipulata*.

**Absent in *C. brasiliense.*

***Only three copies in *M. exstipulata*.

**Figure 2:**
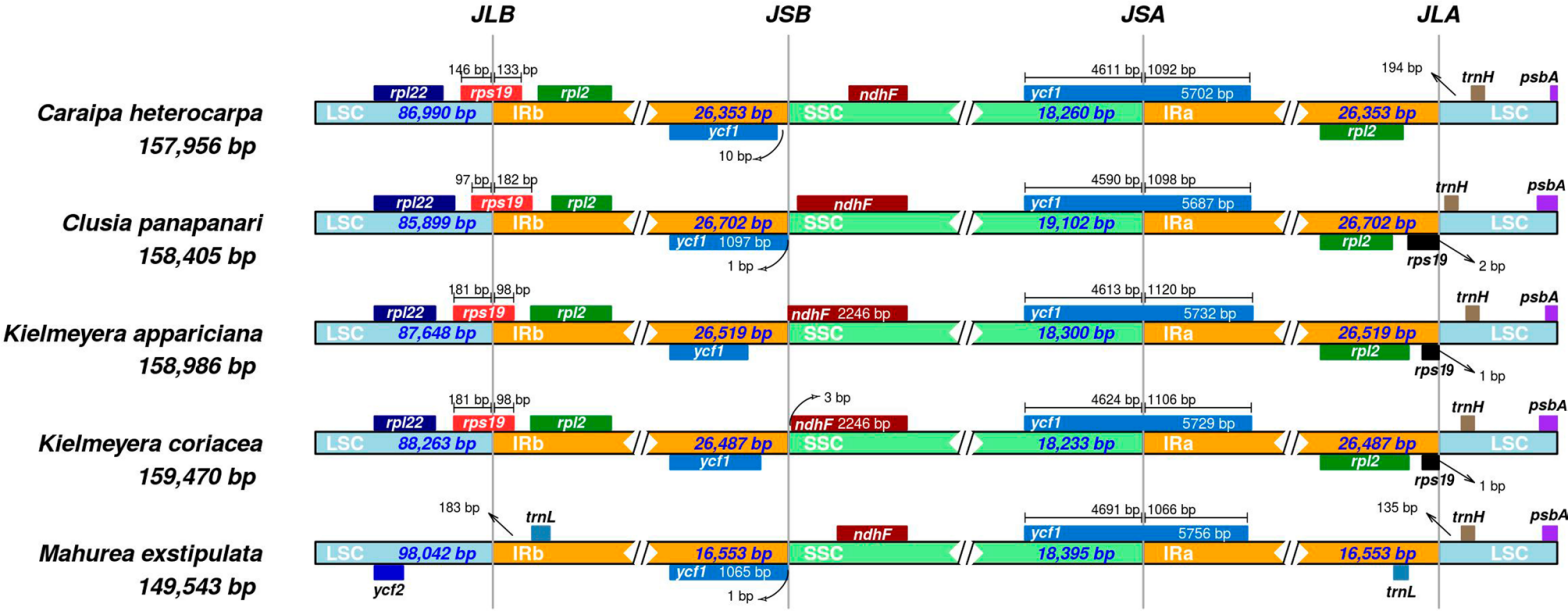
Comparison of the genes flanking the limits of LSC, SSC, and IR regions from the five complete clusioid plastomes assembled. JLB (IRb/LSC), JSB (IRb/SSC), JSA (SSC/IRa) and JLA (IRa/LSC) denote the respective limit in the genome.

Few differences in the limits of the four regions of the plastomes were observed (Fig. 2). The LSC-IRb limit is flanked by *rpl*22 on the LSC side and by *rpl*2 on the IRb side, with *rps*19 spanning the junction in all species but *M. exstipulata*. This species lacks *rps*19 in this region and has *ycf*2 flanking the junction on the LSC side and *trn*L on the IRb side. The IRb-SSC junction always has the *ycf*1-fragment on IRb and the *ndh*F on SSC, while *ycf*1 spans the SSC-IRa junction. Finally, the IRa-LSC junction is flanked by *rpl*2 and *rps*19 on the IRa side and by *trn*H on LSC for all species, although *trn*L is on the IRa side in *M. exstipulata*.

**Figure 3:**
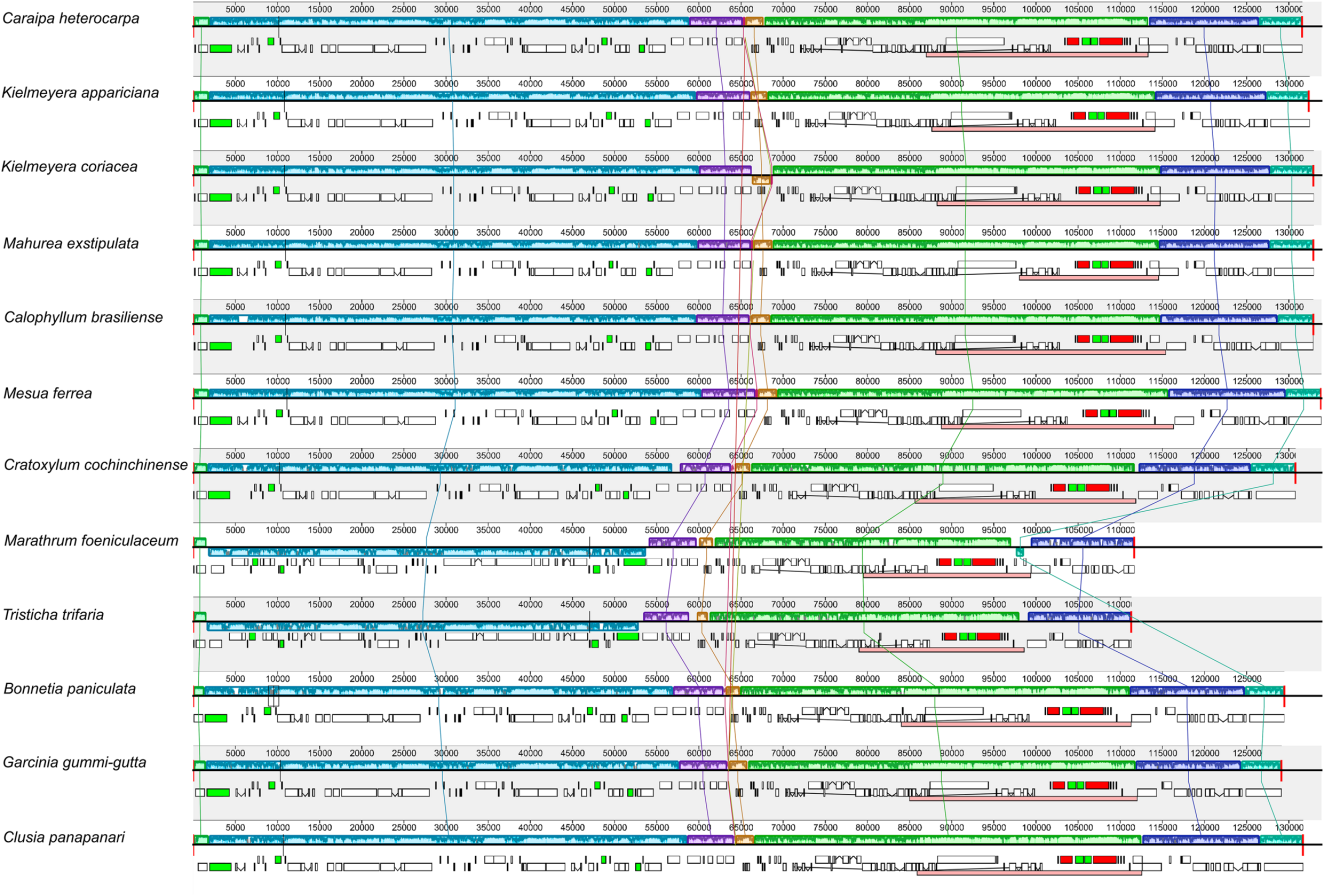
Progressive Mauve alignment showing synteny and rearrangements for twelve clusioid plastomes. Brownish-orange bars represent the *psb*J–*psb*E inversion in *Kielmeyera coriacea* and light blue bars represent the *trn*K–*rbc*L inversion in the two Podostemaceae species (*Marathrum foeniculaceum* and *Tristicha trifaria*).

Mauve alignment of the 12 clusioid plastomes recognized seven synteny blocks and confirmed the *trn*K-UUU–*rbc*L inversion in both Podostemaceae species. Another inversion (*psb*J–*psb*E) was observed only in *K. coriacea*. There were no other major structural differences between the assessed plastomes (Fig. 3).

### Long and simple sequence repeats (SSRs) and sequence polymorphism

REPuter^44^ identified between 20 long repeats in *Tristicha trifaria* and 50 in *Cratoxylum cochinchinense* (Lour.) Blume most of them were forward and palindromic repeats; complementary repeats were found only in *Garcinia gummi-gutta* (L.) N.Robson, which has two of them (Table 3). We found a repeat flanking *rbc*L in *Marathrum foeniculaceum* and a repeat flanking *trn*K in *T. trifaria*, each gene being at one end of the 50 kb inversion reported for the family. No repeats were found flanking the *psb*J–*psb*E inversion in *K. coriacea*, although they are often associated with genomic rearrangements^17^. In almost all clusioid representatives, most long repeats were distributed throughout the LSC, the only exceptions were *B. paniculata* with 15/32 repeats in the LSC and *C. cochinchinense* with 21/50. The number of long repeats in the SSC ranged from one in *T. trifaria* to eight in *M. ferrea*, and in the IR it ranged from three in *M. foeniculaceum* to 31 in *C. cochinchinense* (Supplementary Figure S1). The location of most of those long repeats regarding coding and noncoding regions were between genes, in the intergenic spacers. In our study, repeats were also found within genes (*acc*D, *ccs*A*, ndh*G, *psa*A, *psa*B, *rps*18, *ycf*1, and *ycf*2, and in the trnS-GCU, -UGA, and -GGA), and in some introns (*clp*P, *ndh*A, *ndh*B, *pet*B, *pet*D, *rps*16, and *ycf*3) (Supplementary Table S1). The presence of repeats in these genes was already noted in other studies^45–47^. MISA^48,49^ identified between 297 SSRs in *Caraipa heterocarpa* and 403 in *M. foeniculaceum*, most were mononucleotide A or T repeats. The two Podostemaceae species had fewer dinucleotide SSRs than all other species with 25 in *T. trifaria* and 28 in *M. foeniculaceum*. Tri- and tetranucleotide SSRs were found in all species. Pentanucleotide SSRs were found in almost all species (9/12), and hexanucleotide SSRs were found only in five species (Table 3).

**Table 3:** Comparison of the number of simple sequence repeats (SSRs) and of long repeats present in 12 clusioid species.

Species	Microsatellites (SSRs)							Repeats			
	Mono	Di	Tri	Tetra	Penta	Hexa	Total	Complementary	Forward	Palindromic	Reverse
*Bonnetia paniculata*	358	49	2	6	0	0	415	0	15	13	4
*Calophyllum brasiliense*	306	35	6	8	5	1	361	0	19	10	4
*Caraipa heterocarpa*	297	53	7	5	3	1	366	0	19	14	6
*Clusia panapanari*	337	42	6	10	1	0	396	0	9	18	1
*Cratoxylum cochinchinense*	380	45	3	11	5	0	444	0	40	10	0
*Garcinia gummi-gutta*	321	43	6	9	1	0	380	2	24	21	0
*Kielmeyera appariciana*	300	53	3	10	1	1	368	0	19	8	4
*Kielmeyera coriacea*	304	53	2	8	0	0	367	0	26	11	3
*Mahurea exstipulata*	301	48	4	6	1	0	360	0	13	13	2
*Marathrum foeniculaceum*	403	28	5	14	2	2	454	0	6	15	4
*Mesua ferrea*	309	41	7	8	4	1	370	0	17	17	3
*Tristicha trifaria*	373	25	2	2	0	0	402	0	8	10	2

Sequence polymorphism analyses indicated that the ten longest regions are equally distributed (four in IR, three in LSC and three in SSC) and that eight of them are CDSs. Nine of the ten regions with more segregating sites and more estimated mutations were the same; at least eight of them are CDSs, and six of them are in the LSC. All the ten regions with highest nucleotide diversity were intergenic spacers and seven out of ten are in the LSC. Overall, LSC and SSC had higher variability (nucleotide diversity between 2.9 and 3.5-fold, respectively) when compared to the IRs (Supplementary Table S2).

### Phylogenetic analyses

Our 59 sequences complete alignment prior to the removal of poorly aligned regions on Gblocks^50^ (nogb) has 77,015 bp, including 34,595 distinct patterns, 21,558 parsimony-informative sites, and 21.16% of gaps/missing data. The alignment processed on Gblocks^50^ (gb) has 66,671 bp, including 30,582 distinct patterns, 19,548 parsimony-informative, and 12.94% gaps/missing data. We used three different methods (maximum-likelihood—ML, Bayesian inference—BI, and multispecies coalescent—MC) and eight different datasets (CU: 82 protein-coding genes concatenated unpartitioned—“supergene approach”, CP: 82 protein-coding genes partitioned with individual evolutionary models, 82: a single consensus gene tree per locus used as input, 2050: 25 consensus gene trees from independent runs per locus used as input; each of these four datasets have two versions: one without removal of poorly aligned regions—nogb, and one after removal using Gblocks^50^—gb) in our analyses.

The trees we obtained exhibited the same topology for most relationships. In all analyses the clusioids were recovered in a strongly supported monophyletic clade with all the five families also being monophyletic. Calophyllaceae was recovered sister to a clade composed by Podostemaceae and Hypericaceae. The position of Bonnetiaceae and Clusiaceae changed in different analyses and datasets. Within Calophyllaceae most relationships were stable with the two *Kielmeyera* species grouped in a clade, *Caraipa heterocarpa* being sister to this clade; *Mahurea exstipulata* was recovered sister to *Kielmeyera* + *Caraipa* clade, and *Mesua ferrea* and *Calophyllum* formed another clade. The position of *Mammea americana* was not stable and will be discussed below. Within Podostemaceae, *Tristicha trifaria* was recovered sister to the clade *M. foeniculaceum* + *Podostemum ceratophyllum*. In Hypericaceae, *Cratoxylum cochinchinense* was recovered sister to the clade *Vismia ferruginea* + *Hypericum*; relationships within *Hypericum* were not stable. In Clusiaceae, the two *Clusia* species included in the study grouped in a clade which was recovered sister to the clade including the two *Garcina* species. The two Bonnetiaceae genera, represented by *B. paniculata* and *Ploiarium* sp., were recovered in a clade. All stable branches mentioned above received 100% (ultrafast bootstrap—UB)/1 (posterior probability—PP) support values.

**Figure 4:**
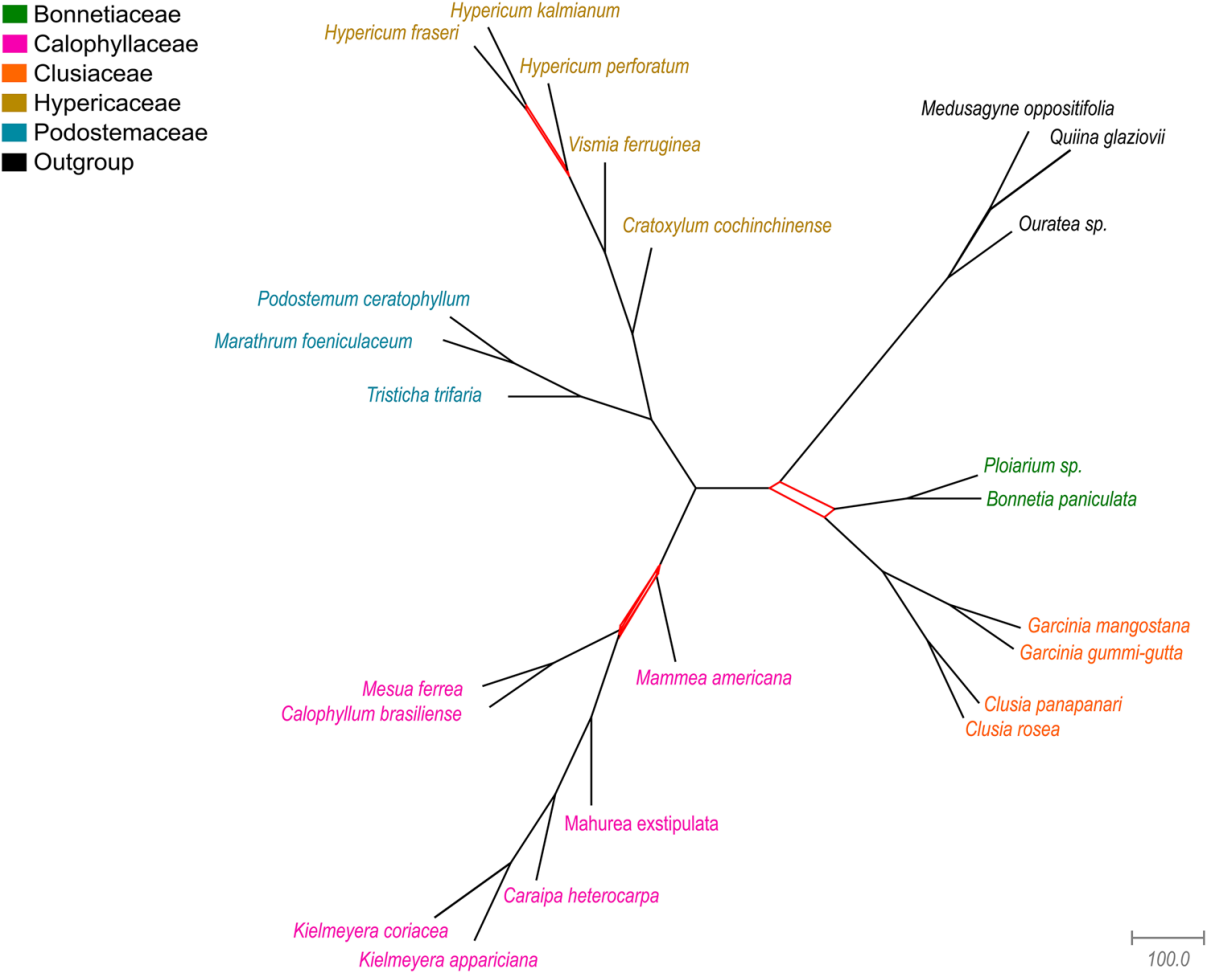
Network representation of the relationships within the clusioid families. Compatible sets of splits are represented by a single branch, and splits where there is incompatibility are represented by a band of parallel branches, colored in red. Families are colored following the legend; names in black denote the outgroup.

**Figure 5:**
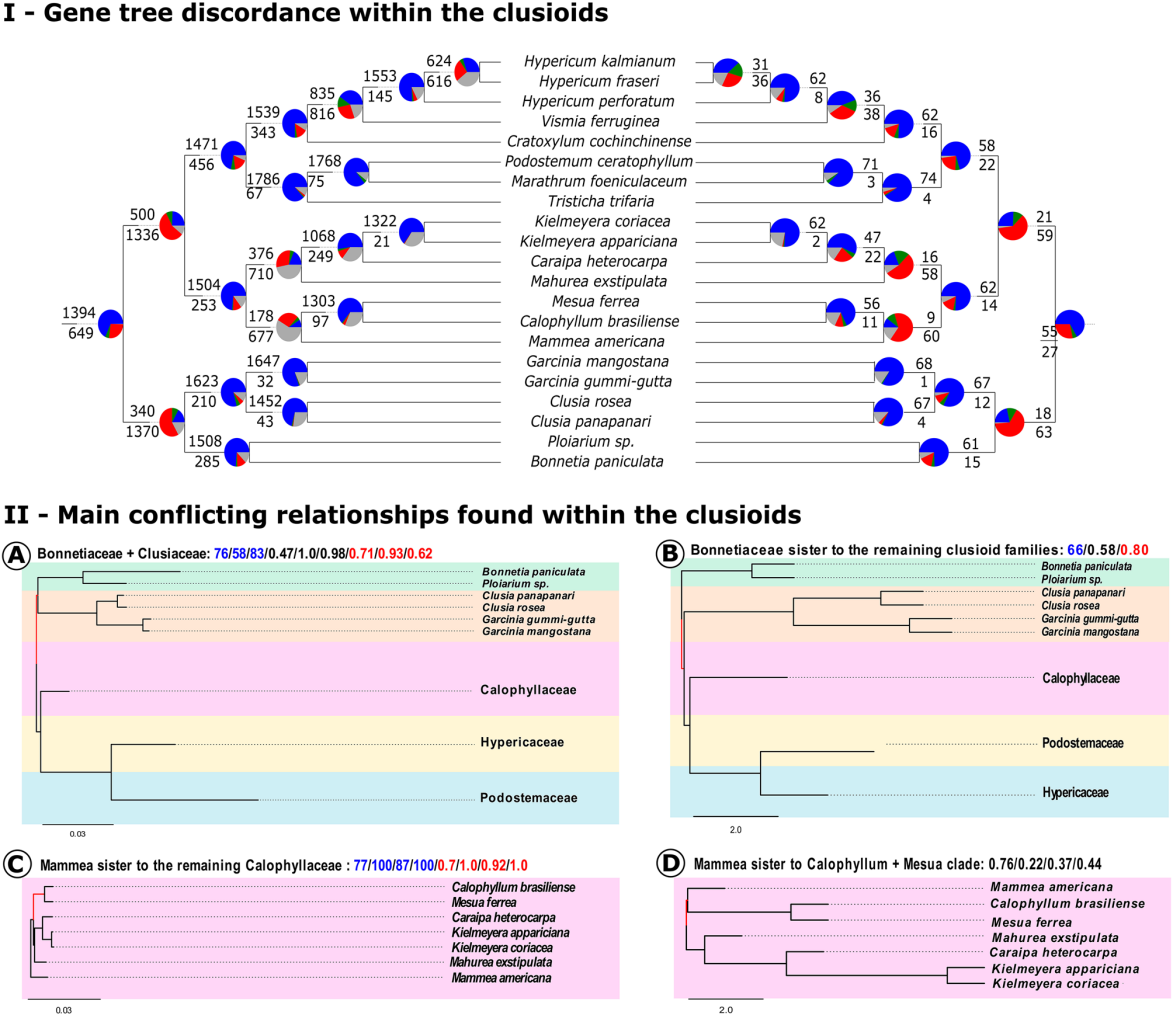
Relationships within the clusioid families and summary of conflicts. On the top, (I) Gene tree discordance within the clusioids represented in the coalescent-based species tree. Pie charts summarize the proportion of conflicting (red and green), concordant (blue) and non-informative (gray) genes for each branch. Numbers above branches indicate concordant genes at that node, and below conflicting genes. On the right the tree generated with one tree for each of the 82 genes (82), and on the left the tree generated with 25 independent replicates for each of the 82 genes (2050), both after removal of poorly aligned sequences (gb). On the bottom, (II) Summary of the main conflicting relationships recovered using maximum-likelihood (values in blue), Bayesian inference (values in red), and multispecies coalescent (values in black) and eight different datasets (CU: 82 protein-coding genes concatenated unpartitioned, CP: 82 protein-coding genes partitioned with individual evolutionary models, 82: a single consensus gene tree per locus used as input, 2050: 25 consensus gene trees from independent runs per locus used as input; each of these four datasets have two versions: one without removal of poorly aligned regions–nogb, and one after removal using Gblocks ^50^–gb). On the top of each tree there are support values for the branch highlighted in red. The two upper trees (**A** and **B**) represent alternative topologies for the position of Bonnetiaceae and Clusiaceae families. The two bottom trees (**C** and **D**) represent alternative topologies for the position of *Mammea americana* (Calophyllaceae). Support values are represented by ultrafast bootstrap (UB) or posterior probability (PP), and the respective dataset (CUgb, CUnogb, CPgb, CPnogb, 82gb, 82nogb, 2050gb, 2050nogb) are in parenthesis. A. Bonnetiaceae sister to Clusiaceae: 76% (UB, CUgb)/58% (UB, CPnogb)/83% (UB, CPgb)/0.47 (PP, 82gb)/1.0 (PP, 2050gb)/ 0.98 (PP, 2050nogb)/0.71 (PP, CUgb)/0.93 (PP, CPgb)/ 0.62 (PP, CPnogb). B. Bonnetiaceae sister to the other clusioid families: 66% (UB, CUnogb)/0.58 (PP, 82nogb)/0.80 (PP, CUnogb). C. *Mammea americana* sister to the other Calophyllaceae species: 77% (UB, CUgb)/ 100% (UB, CUnogb)/ 87% (UB, CPgb)/ 100% (UB, CPnogb)/0.7 (PP, CUgb)/1.0 (PP, CUnogb)/0.92 (PP, CPgb)/1.0 (PP, CPnogb). D. *Mammea americana* in a clade with *Calophyllum brasiliense* and *Mesua ferrea*: 0.76 (PP, 2050gb)/0.22 (PP, 2050nogb)/0.37 (PP, 82gb)/0.44 (82PP, nogb).

The three relationships that changed based on the analyses type and on the dataset were: (1) the position of the Bonnetiaceae and Clusiaceae, (2) the positions of *Mammea* (Calophyllaceae), and (3) the relationship between *Hypericum fraseri* Steud. and *H. kalmianum* L. (Hypericaceae). These relationships are represented in the network (Fig. 4). Conflicting relationships from 1 and 2 are summarized in Fig. 5. Filtering the poorly aligned regions, using partitioned schema, and allowing different evolutionary models usually increased support values in analyses and—importantly—changed the topology. For conflicting relationship 1, ML and BI unpartitioned analysis after removal of poorly aligned regions (CUgb), all ML and BI partitioned analyses (CPgb and CPnogb) and most MC species trees (82gb, 2050gb, 2050nogb) recovered Clusiaceae as sister to Bonnetiaceae (Fig. 5-IIA). Only ML and BI unpartitioned analysis without removal of poorly aligned regions (CUnogb), and MC species tree using a single gene tree per gene as input without removal of poorly aligned regions (82nogb) recovered Bonnetiaceae as sister to the remaining clusioid families (Fig. 5-IIB). Support values for this relationship were generally low to moderate. For conflicting relationship 2, all ML and BI consensus trees regardless of partitioning scheme or removal of poorly aligned regions (CUgb, CUnogb, CPgb, CPnogb) recovered *Mammea* as sister to the remaining Calophyllaceae (Fig. 5-IIC). All MC species trees (82gb, 82nogb, 2050gb, 2050nogb) recovered *Mammea* as sister to the clade *Mesua* + *Calophyllum*, and this clade as sister to a clade including the remaining Calophyllaceae (Fig. 5-IID). In all ML and BI analyses, support values for this relationship were moderate to high whereas they were low in MC analyses. Relationship 3 will not be discussed since few samples from the genus *Hypericum* L. were included here (3/370 species). PhyParts^51^ gene tree discordance analyses using MC trees after removal of poorly aligned regions (82gb, 2050gb) as input showed that the two of three branches with lower support in our analyses are the ones where there is more gene conflict (Fig. 5-I). Particularly for taxa in Calophyllaceae, around 50% of the genes were not informative or resulted in conflicting topologies. The discussion regarding gene tree discordance will be based on MC species trees generated using the datasets with poorly aligned regions removed (82gb and 2050gb).

## Discussion

Five out of the six newly assembled plastomes (including the almost complete plastome of *Calophyllum brasiliense*) presented here showed a conserved structure with total size, general organization in a quadripartite structure, IR, LSC and SSC sizes, number of genes and GC content in agreement with the values for an ordinary angiosperm plastome^7,17^. However, as is the case in several groups, our study focusing on Calophyllaceae revealed that there is variation of plastome structure for the family. *Mahurea exstipulata* chloroplast had only a single copy of the usually duplicated genes *rpl*2, *rpl*23 and *ycf*2, which moved from the IRs to the LSC, resulting in the contraction of former and the expansion of the latter and in distinct IR/single-copy regions junctions. The expansion or contraction of IRs is a common type of rearrangement in plastomes that was already documented for the clusioids in Podostemaceae^39,40^ and for some other groups like Geraniaceae^52^, Acanthaceae^53^ and the genus *Passiflora*^46^. However, this phenomenon was not observed in Calophyllaceae until the present study. Although Podostemaceae species and *M. exstipulata* share IR contractions, their plastomes are quite distinct and these contractions represent independent evolutionary events. In *M. exstipulata* the IR contraction resulted from the loss of one copy of the genes *rpl*2, *rpl*23 and *ycf*2 and it was associated with a large expansion of the LSC; it is between 9,258 and 19,040 bp longer when compared to the other clusioids. The loss of a copy of the same three genes is a rare event and has been reported only in *Strobilanthes cusia* (Nees) Kuntze (Acanthaceae, Lamiales)^53^, and the loss of *rpl*2 and *rpl*23 is known from *Cuscuta reflexa* Roxb. (Convolvulaceae, Solanales)^54^ and *Lonicera japonica* Thunb. (Caprifoliaceae, Dipsacales)^55^ On the other hand, in Podostemaceae the IR contraction resulted from both gene (*ycf*1 and *ycf*2) and intron (*rps*16) loss and pseudogenization of *acc*D, *rpl*22, and *clp*P, all associated with a reduction also in the single copy regions, which resulted in a smaller chloroplast as a whole. Among the newly sequenced plastomes, all species but *M. exstipulata* had the junctions between IR and LSC and SSC regions similar to those of a canonical angiosperm plastome^56^. The differences observed in the limits of the four main parts of the plastome correspond to one of the sources of variation in the plastome structure and have been described from different taxonomic levels, e.g., within Calophyllaceae and between the clusioids or within Cercidoideae legumes^56^. The usually slower evolutionary rates of genes IRs when compared to single copy regions is widely reported in the literature^14,57^ and was also confirmed in our study. Regarding genes and intergenic spacers, the latter were the most variable and should be considered in future evolutionary studies. In agreement with Walker and collaborators (2019)^25^, the *rpoC*2 was the gene with more segregating sites.

Regarding other structural differences noted in the clusioid chloroplasts, the *trn*K-UUU–*rbc*L inversion was first noted for Podostemaceae by Bedoya et al. (2019)^39^ and Jin et al. (2020)^40^. Although Jin et al. (2020)^40^ also reported this inversion for *Cratoxylum cochinchinense* (Hypericaceae) and suggested it could be a synapomorphy of the clade composed by these two families, it was observed only in Podostemaceae in our study. We also registered the first inversion within Calophyllaceae (*psb*J-*psb*E) in *Kielmeyera coriacea*. Repetitive regions are known to flank inversion breakpoints. In our data, it was observed only in Podostemaceae (Supplementary Table 1). The *ycf*3 intron loss has not been reported for any other angiosperm except the clusioids to our knowledge and could be a synapomorphy of the clade but further investigation of the chloroplast structure of the closely related families is necessary to confirm this hypothesis. We speculated that the intron 2 was lost from *ycf*3, since in Petersen et al. (2011)^58^ the loss of intron 1 from that gene in tobacco resulted in phenotypically mutant plants and prevented the splicing of intron 2.

Phylogenetic relationships within the clusioid families are frequently reported as challenging and historically they have changed quite a lot. Savolainen et al. (2000)^59^, Chase et al. (2002)^60^ and Gustafsson et al. (2002)^61^ all used *rbc*L to infer relationships and recovered Podostemaceae (P) sister to Hypericaceae (H) and Calophyllaceae (Ca) sister to Clusiaceae *s.s.* (Cl) with Bonnetiaceae (B) as sister to the Podostemaceae + Hypericaceae clade (Savolainen et al. 2000, Chase et al. 2002)^59,60^ or as sister to a clade including all the other four families (Gustafsson et al. 2002)^61^. In all three studies, support values for the position of Bonnetiaceae and for Clusiaceae + Calophyllaceae were moderate to low and the monophyly of the clusioids was poorly supported. A different topology was proposed by Wurdack and Davis (2009)^62^ who analyzed 13 genes from all three compartments and recovered ((Cl + B) + (Ca (H + P))) and confirmed clusioid monophyly. Similarly, Soltis et al. (2011)^63^ recovered different relationships using 17 genes, most of them from the chloroplast: (((P + H) + Ca) + B) + Cl)). In the same year, Ruhfel et al. (2011)^35^ using three plastid and one mitochondrial genes and the most extensive sampling of the clade so far (194 species included), recovered the same topology of Wurdack and Davis (2009)^62^ as did Xi et al. (2012)^33^ using genes from the three genomic compartments and Jin et al. (2020)^40^ using 82 protein-coding plastid genes. More recently, Cai et al. (2021)^34^ recovered a distinct topology using a 423 nuclear gene matrix: (((Ca + B) + H) + Cl)-Podostemaceae were not included, however, there is much incongruence between gene trees and the species tree. Our results mostly agree with the topology from Wurdack and Davis (2009)^62^, but also reveal much gene tree disagreement, particularly for two relationships: (1) Clusiaceae + Bonnetiaceae that is supported by only 340 of the total 2050 gene trees and 18 of the total 82 gene trees, and (2) Calophyllaceae + (Hypericaceae + Podostemaceae) is supported by 500 of the total 2050 gene trees and 21 of the total 82 gene trees (Fig. 5-I). Interestingly, a position of Bonnetiaceae as sister to the rest of the clusioid clade was proposed by Engler^64^ and mainly supported by the lack of the schizogenic latex or resin ducts in Bonnetiaceae. However, this position was not recovered in any recent molecular phylogeny until the current study and in Baker et al*.*^65^, but in the latter Podostemaceae was not with the other clusioids.

Relationships within Bonnetiaceae, Clusiaceae, and Podostemaceae are largely in agreement with other studies^30^, *Clusia* and *Garcinia* L. being the only genera with more than one representative and recovered as monophyletic in all our analyses. Within Hypericaceae, our results recovered the same relationship as found by Ruhfel et al. (2016)^41^, with *Cratoxylum cochinchinense* (Cratoxyleae) + (*Vismia ferruginea* Kunth. (Vismieae) + *Hypericum* spp. (Hypericeae)). Within Calophyllaceae, only representatives from the tribe Calophylleae were included in the present study. Ruhfel et al. (2011, 2016)^35,41^, recovered *Mammea* in a clade with *Kayea* and *Poeciloneuron*, and this clade was sister to a clade including *Calophyllum* and *Mesua*. Recently, Cabral et al. (2021)^42^ evaluated relationships between Calophylleae genera. The authors recovered *Mammea* in a clade with *Poeciloneuron* alone and this clade as sister to a large clade including the genera *Calophyllum*, *Caraipa*, *Clusiella*, *Haploclathra*, *Kielmeyera*, *Marila*, *Mahurea*, and *Mesua*, although with low support. Our analyses recovered both positions for *Mammea*. *Mammea* was recovered as sister to the clade *Calophyllum* + *Mesua*, as in Ruhfel et al. (2011, 2016)^35,41^, in all four coalescent schema, i.e., 82gb, 82nogb, 2050gb and 2050nogb, but all with low support (PP 0.37, 0.44, 0.76 and 0.22, respectively) (Fig. 5-IID). The high level of uninformative gene trees (uninformative gene trees/total number of gene trees: 1195 /2050, 13/82) associated with a considerable number of discordant trees (discordant trees/total number of trees: 677/2050, 60/82) may help to explain the low support observed (Fig. 5-I). In all four ML and BI concatenated analyses (CUgb, CUnogb, CPgb, CPnogb), *Mammea* was recovered as sister to a large clade including the other genera, as in Cabral et al. (2021)^42^, with high support (100% UB/1.0 PP), and the relationship between the clade (*Mesua* + *Calophyllum*) and the clade with the remaining genera was moderately to strongly supported (UB/PP: CUnogb: 100%/1.0, CUgb: 77%/0.7, CPnogb: 100%/1.0, CPgb: 87%/0.92) (Fig. 5-IIC).

The conflicts reported here show that partitioning the dataset, filtering for poorly aligned regions and the method of inference can impact the topology of the tree. The use of MSC, considering each plastid gene individually, was recently recommended to infer phylogenies with plastid data^22^; this approach breaks some assumptions of the model (see^24^ for a discussion). However, despite scarce, there is evidence of recombination in chloroplasts [see ^25^,^66^], raising a question that deserves further exploration. Thus, it would be interesting to see studies testing for non-recombinant units in the plastid prior to phylogenetic inference. There is a lot to be learned about organellar genomes, including a deeper investigation of its multibranched structure.

Plastid data can be a good starting point to investigate phylogenetic relationships, particularly in large and neglected genera such as* Clusia* and *Calophyllum*, with around 300–400 and 200 species, respectively. Whenever possible, it should be combined with data from other genomic compartments. Obtaining genomic DNA from herbarium specimens to target nuclear regions is still challenging, although it may become more common with the development of probe sets that can be used in different groups and of standardized protocols^16^. In this scenario, chloroplast DNA data availability will also increase since this information is being recovered with nuclear DNA in target enrichment capture due to the presence of plastids in high copy number in a cell. Furthermore, an initial exploration may help to point out groups where more sampling is needed or where relationships will need a different source of data to be clarified.

The disagreements found in this study have been reported since the first molecular phylogenies for the clusioids appeared. We now need to add data from other genomic compartments (i.e., nucleus and mitochondria). However, what Cai et al. (2021)^34^ have shown with nuclear data is that reconstructing phylogenies in Malpighiales, to which the clusioids belong, represents a huge challenge due to incomplete lineage sorting, tree error estimation and gene flow. Therefore, for a better comprehension of phylogenetic relationships, more analytical refinement is required, such as testing different partitioned schema.

## Methods

### Taxon Sampling, DNA extraction and sequencing

Silica dried leaves of *Kielmeyera appariciana*, *K. coriacea*, *Caraipa heterocarpa* and *Mahurea exstipulata* were sampled during the development of R. J. Trad and F. N. Cabral theses and vouchers have been deposited in the University of Campinas herbarium (UEC). All the plants included in the study were collected with ICMBio fieldwork permits (n. 23954–4 and 41896–3 to R.J.T. and n. 33308 to F.N.C.), and INPA permit to collect in the Ducke Reserve for F.N.C. Collections and experimental research on the plants present in the study complied with international, national, and/or institutional guidelines. *Kielmeyera* specimens (Trad 192—*K. appariciana* and Trad 401—*K. coriacea*) were identified by Rafaela Jorge Trad and *Caraipa* and *Mahurea* specimens (Cabral FC705—*C. heterocarpa* and Cabral FC1140—*M. exstipulata*) were identified by Fernanda Nunes Cabral. A sample of *Calophyllum brasiliense* was obtained from the specimen N. Hind 4260 from UEC herbarium; its identification was confirmed by Maria do Carmo Estanislau do Amaral and Volker Bittrich. Reads of *Clusia panapanari* were downloaded from GenBank (accession number SRR7518735). Complete voucher information is presented in Supplementary Table S3.

Total DNA of six samples was extracted following Doyle & Doyle^67^ modified by Caddah^68^. The DNA was quantified on NanoDrop Spectrophotometer (Thermo Scientific, Waltham, MA, USA) or on Qubit 2.0 (Invitrogen, Carlsbad, CA, USA). Genomic libraries for whole-genome were prepared and sequenced 2 × 150 bp on Illumina NextSeq 500 Mid-Output by Genohub (Austin, TX, USA). All adapters were removed by the company.

### Genome assembly and annotation

Reads were assembled in two ways. (1) Using GetOrganelle v.1.6.4^11^ with the following parameters: -R 15 -k 21,35,45,55,65,75 –max-reads 4E7 -F embplant_pt. All assembly graphs from GetOrganelle^11^ were visually checked in Bandage v 0.8.1^69^, so parameters could be adjusted. For all species two configurations of the plastome were assembled. For *Calophyllum brasiliense* and for *Kielmeyera coriacea*, plastomes were assembled in a different way. (2) Reads were mapped to the closest chloroplast available at the time this study started, i.e., *Garcinia mangostana* L. (GenBank accession NC_036341.1) with Bowtie2 v 2.2.1^70^ plugin on Geneious 9^12^ by adding the command *–no-discordant* to the default parameters; mapped reads were assembled with Platanus v.1.2.4^71^. Since the two haplotypes found are present in the same proportion in a cell^28,72^, we chose the same configuration of the plastome published for *G. mangostana* to use in subsequent analysis. To validate our assemblies and to verify the coverage, reads were mapped to the assembled genomes using Bowtie2 v 2.2.1^70^ as mentioned above but using the assembled plastome as reference. Between 49,148 and 540,562 reads mapped the respective assembled chloroplast (Table 1).

Assembled chloroplasts were automatically annotated using GeSeq^73^ implemented on Chlorobox website (https://chlorobox.mpimp-golm.mpg.de/). The annotation was checked and corrected on Geneious 9^12^. Start and stop codons were checked for all the genes and tRNAs limits were checked using ARAGORN^74^ output from Chlorobox (https://chlorobox.mpimp-golm.mpg.de/). Potential pseudogenes were defined by Blast following Silva et al. (2018)^75^. Finally, circular gene maps were generated with OGDRAW^76^. All subsequent analyses, except IRscope^77^, were performed on plastomes with only one IR to avoid redundant results.

### Plastome structure and identity

The gene content was compared between the five Calophyllaceae and the Clusiaceae newly assembled plastomes (Table 2). For general plastome structure and all phylogenetic analyses, all the annotated and checked plastomes available for the clusioid clade on GenBank were included: *Bonnetia paniculata* Spruce ex. Benth. (Bonnetiaceae), *Mesua ferrea* L. (Calophyllaceae) *Garcinia gummi-gutta* (Clusiaceae), *Cratoxylum cochinchinense* (Hypericaceae), *Marathrum foeniculaceum* (Podostemaceae) and *Tristicha trifaria* (Podostemaceae). All the GenBank accession numbers are found in Table 1. We manually adjusted the beginning of each plastome on Geneious 9^12^. Beginning in the same region is important to avoid Mauve infer false rearrangements. A total of 12 plastomes were aligned using progressive Mauve algorithm in Mauve Plugin v. 2.3.2 in Geneious 9^12^ to check for structural differences such as inversions or rearrangements. IR boundaries were evaluated on IRScope online^77^.

### Repetitive regions and polymorphism

Since reorganizations can be associated with small dispersed repeats^17^, REPuter^44^ was used to identify direct, complement, palindromic, and reverse repeats with the following parameters: minimal size of 30 pb and Hamming distance of 3. For *Calophyllum brasiliense* and for *Kielmeyera coriacea* the Ns and IUPAC ambiguous bases had to be manually removed from the respective fasta file in order to run REPuter^44^. And MISA^48,49^ was used to identify simple sequence repeats (SSR) with a minimum number of 7, 4, 4, 3, 3, and 3, for mono-, di-, tri-, tetra-, penta-, and hexanucleotide repeats, respectively. The parameters followed Silva et al. (2019)^78^.

To evaluate sequence polymorphism, protein-coding and intergenic regions were extracted with parseGenbank.pl script from Mitofy^79^ or on Geneious 9^12^ and aligned on MAFFT v. 7.308^80^ with the ‘adjustdirection’ option added to the default parameters. Only intergenic regions longer than 50 bp were included and *Azara serrata* Ruiz & Pav. (Salicaceae) was included in the alignments as an outgroup. Alignments were used as input on DnaSP 6^81^ to calculate variation between regions. We compared total number of sites, of analyzed positions (NetSites), of segregating sites, of conserved sites, the estimated number of mutations, parsimony informative sites (PIS), proportion of PIS, nucleotide diversity and average number of substitutions per site. The proportion of PIS was calculated as the PIS/NetSites × 100 and gives an estimate about the absolute informativeness of the region. This information, when combined with the length of the region, may be helpful for markers design.

### Phylogenetic analysis

For the phylogenetic analyses we assembled a dataset with the 82-protein-coding genes that includes two Bonnetiaceae species (two genera), seven Calophyllaceae species (six genera), four Clusiaceae species (two genera), five Hypericaceae species (three genera) and three Podostemaceae species (three genera). Additionally, data for other 36 species within Malpighiales, *Averrhoa carambola* L. (Oxalidales) and *Elaeodendron orientale* Jacq. (Celastrales) were also included as outgroups. A list with all the GenBank accession numbers for additional species is included in the Supplementary Material S1. To assure poorly aligned regions were not interfering in tree topology, these regions were removed with Gblocks^50^ with the following parameters: -t = d -b5 = a -n = y -e = gb1 -d = y. Therefore, the following analyses were conducted both for the original dataset (nogb) and for the dataset after gblocks (gb). Maximum likelihood inference was conducted based on the concatenated approach for the unpartitioned (CUnogb and CUgb) and partitioned datasets (CPnogb and CPgb) in IQ-tree 2^82^. Models were selected based on Bayesian information criterion (BIC) and support was assessed through 1000 ultrafast bootstraps. For both the nogb and the gb alignments, a total of 25 independent runs were performed for each gene, totaling 2050 consensus gene trees which were included in a single file to generate the 2050 datasets, i.e., 2050nogb and 2050gb. Bayesian Inference for the unpartitioned (CUnogb and CUgb) and partitioned datasets (CPnogb and CPgb) was conducted in Mr.Bayes v.3.2.7a under the best-of-fit model in accordance with BIC assessed in IQ-tree2^82,83^. It was run for 30 million generations sampled every 1000 generations, using two runs and four chains, until the average standard deviation of split frequencies became less than 0.01, beginning with random trees. The initial trees were discarded after reaching stationarity (~ 25%). Also, a coalescent-based species tree estimation was done in Astral-III^84^. We conducted the analysis with one tree per gene as input (82gb and 82nogb) and with 25 trees per gene as input (2050gb and 2050nogb). All gene trees used as input in Astral-III^84^ were rooted and had branches with support lower than 10% collapsed in Newick Utilities^85^ through the functions nw_reroot and nw_ed. Rooting the trees and collapsing branches with low support are known to improve the performance of summary methods^86^.

To explore tree conflict within the clusioid families the splits.nex file generated in IQ-tree2^82^ for both partitioned and unpartitioned analysis was visualized as a network in SplitsTree4 v. 4.16.2^87^. Additionally, tree conflict was explored through PhyParts^51^ and pie charts were plotted on the species phylogeny using the PieCharts python script developed by M. Johnson^88^. PhyParts calculates the number of concordant gene trees, of the top alternative bipartition, of other conflicting topologies, and of uninformative genes for all branches in the tree. To root the trees and remove ultrafast bootstrap and branch length values the files for both PhyParts^51^ and PieCharts were prepared using Newick Utilities^85^ through the functions nw_reroot and nw_topology.

## Supplementary Information

Supplementary Information.

## Data Availability

The complete plastome sequences of *Calophyllum brasiliense*, *Caraipa heterocarpa*, *Kielmeyera appariciana*, *K. coriacea*, and *Mahurea exstipulata* have been submitted to GenBank under the accession numbers MW853786MW853790.
