# Supplementary material for: Calophyllaceae plastomes, their structure and insights in relationships within the clusioids
Source: Sci Rep. 2021 Oct 20;11:20712. doi: 10.1038/s41598-021-99178-z (PMC8528878; doi:10.1038/s41598-021-99178-z)
Supplement: Supplementary file 1 — Supplementary Information. [file 41598_2021_99178_MOESM1_ESM.pdf]

Supplementary Figure

Title: Calophyllaceae plastomes, their structure and insights in relationships within the clusioids

Authors: Rafaela Jorge Trad*, Fernanda Nunes Cabral, Volker Bittrich, Saura Rodrigues da Silva and Maria do Carmo Estanislau do Amaral
*corresponding author: rafajt@hotmail.com

Supplementary Figure S1. Distribution of long repeats in the three plastome regions (LSC – large single copy, IR – inverted repeat, SSC- small single copy) of 12 clusioid species.

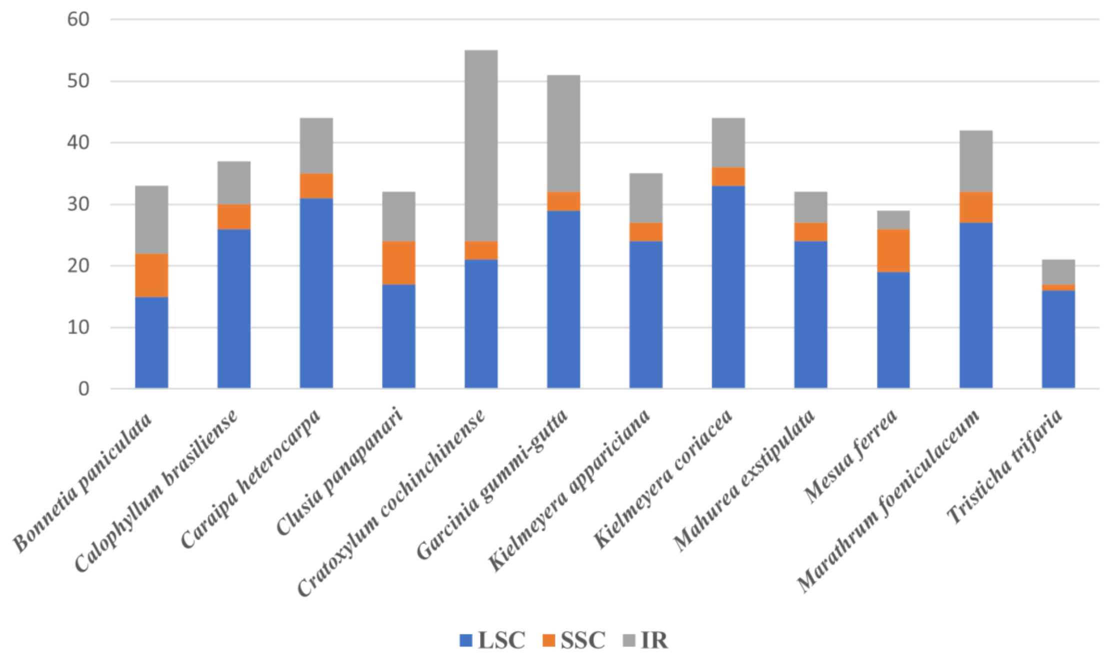

Supplementary Tables

Title: Calophyllaceae plastomes, their structure and insights in relationships within the clusioids

Authors: Rafaela Jorge Trad*, Fernanda Nunes Cabral, Volker Bittrich, Saura Rodrigues da Silva and Maria do Carmo Estanislau do Amaral

*corresponding author: rafajt@hotmail.com

Supplementary Table S1. Summary information about long repeats found in the clusioids plastomes. The location in the plastome is represented only once when both repeats are in the same region; large single copy (LSC), inverted repeat (IR), small single copy (SSC). The number in parenthesis refer the repeat: (1) first repeat and (2) second repeat.

Bonnetia paniculata Spruce ex. Benth.					
First repeat		Second repeat		Orientation	Position in the chloroplast
Lenght	Start position	Length	Start position		
75	91,872	75	91,890	F	*ycf*2 (IR)
66	91,881	66	91,899	F	*ycf*2 (IR)
48	91,881	48	91,917	F	*ycf*2 (IR)
41	39,523	41	41,747	F	*psa*B (1); *psa*A (2) (LSC)
40	99,215	40	121,667	F	between *rps*12-*trn*V-GAC (1) (IR); *ndh*A intron (2) (SSC)
43	91,872	43	91,908	F	*ycf*2 (IR)
39	91,872	39	91,926	F	*ycf*2 (IR)
31	91,916	31	91,934	F	*ycf*2 (IR)
34	100,454	34	100,470	F	between *rps*12 and *trn*V-GAC (IR)
31	91,898	31	91,934	F	*ycf*2 (IR)
30	91,881	30	91,935	F	*ycf*2 (IR)

34	72,128	34	72,130	F	between *clp*P and *psb*B (LSC)
33	27,927	33	27,928	F	between *rpo*B and *trn*C-GCA (LSC)
32	7,223	32	35,966	F	*trn*S-GCU (1); *trn*S-UGA (2) (LSC)
32	84,286	32	84,311	F	between *trn*H-GUG and *rps*19 (IR)
49	20,768	49	20,768	P	between *rpo*C2 and *rpo*C1 (LSC)
50	77,926	50	77,926	P	between *pet*D and *rpo*A (LSC)
51	52,019	51	52,019	P	between *trn*M-CAU and *atp*E (LSC)
34	29,782	34	29,782	P	between *pet*N and *psb*M (LSC)
32	113,952	32	113,952	P	between *ndh*F and *rpl*32 (SSC)
30	63,723	30	63,779	P	between *pet*A and *psb*J (LSC)
37	9,211	37	9,211	P	between *trn*G-UCC and *trn*R-UCU (LSC)
30	7,225	30	45,091	P	*trn*S-GCU (1); *trn*S-GGA (2) (LSC)
30	127,378	30	127,378	P	*ycf*1 (SSC)
31	114,653	31	114,653	P	between *rpl*32 and *trn*L-UAG (SSC)
30	8,006	30	8,038	P	between *trn*S-GCU and *trn*G-UCC (LSC)
30	28,497	30	70,097	P	between *trn*C-GCA and *pet*N (1); *clp*P intron (2) (LSC)
30	35,968	30	45,091	P	*trn*S-UGA (1); *trn*S-GGA (2) (LSC)
39	125,268	39	125,268	R	*ycf*1 (SSC)
31	32,524	31	32,524	R	between *trn*T-GGU and *psb*D (LSC)
31	114,651	31	114,651	R	between *rpl*32 and *trn*L-UAG (SSC)
33	114,191	33	114,195	R	between *ndh*F and *rpl*32 (SSC)

Calophyllum brasiliense Cambess.					
First repeat		Second repeat		Orientation	Position in the chloroplast
Lenght	Start position	Length	Start position		
56	33,598	56	33,653	F	between *trn*T-GGU and psbD (LSC)
41	101,540	41	124,292	F	between *rps*12 and *trn*V-GAC (1) (IR); *ndh*A intron (2) (SSC)
47	40,293	47	42,517	F	*psa*B (1); *psa*A (2) (LSC)

35	13,098	35	13,119	F	between *atp*F and *atp*H (LSC)
38	1,884	38	1,903	F	between *psb*A and *trn*K (LSC)
39	44,600	39	101,542	F	*ycf*3 intron (1) (LSC); between *rps*12 and *trn*V-GAC (2) (IR)
39	44,600	39	124,294	F	*ycf*3 intron (1) (LSC); *ndh*A intron (2) (SSC)
41	94,174	41	94,192	F	*ycf*2 (IR)
32	8,354	32	8,369	F	between *trn*S-GCU and *trn*S-CGA (LSC)
31	130,434	31	130,461	F	*ycf*1 (SSC)
30	46,208	30	46,236	F	between *trn*S-GGA and *rps*4 (LSC)
30	46,916	30	46,942	F	between *rps*4 and *trn*C-UGU (LSC)
34	102,765	34	102,781	F	between *rps*12 and *trn*V-GAC (IR)
31	94,184	31	94,202	F	*ycf*2 (IR)
32	91,741	32	91,762	F	*ycf*2 (IR)
31	69,482	31	69,507	F	between *psa*J and *rpl*33 (LSC)
30	7,569	30	7,582	F	between *trn*S-GCU and *trn*S-CGA (LSC)
30	44,612	30	101,554	F	*ycf*3 intron (1) (LSC); between *rps*12 and *trn*V-GAC (2) (IR)
30	48,241	30	48,252	F	*trn*S-AGA intron (LSC)
56	30,491	56	30,491	P	between *pet*N and *psb*M (LSC)
44	76,570	44	76,570	P	between *psb*T and *pbf*1 (LSC)
41	80,267	41	80,267	P	between *pet*D and *rpo*A (LSC)
32	30,385	32	30,385	P	between *pet*N and *psb*M (LSC)
38	51,696	38	51,696	P	between *ndh*C and *trn*C-ACA (LSC)
30	7,267	30	45,910	P	*trn*S-GCU (1); *trn*S-GGA (2) (LSC)
35	128,926	35	128,926	P	*ycf*1 (SSC)
32	53,547	32	53,547	P	between *trn*M-CAU and *atp*E (LSC)
30	29,824	30	29,824	P	between *pet*N and *psb*M (LSC)
31	32,769	31	32,769	P	between *trn*E-UUC and *trn*T-GGU (LSC)
33	51,585	33	51,585	R	between *ndh*C and *trn*C-ACA (LSC)
31	43,884	31	43,884	R	between *psa*A and *ycf*3 (LSC)

30	7,648	30	74,411	R	between *trn*S-GCU and *trn*S-CGA (1); between *clp*P and *psb*B (2) (LSC)
30	64,701	30	69,588	R	between *pet*S and *psb*J (1); between *psa*J and *rpl*33 (2) (LSC)

Caraipa heterocarpa Ducke					
First repeat		Second repeat		Orientation	Position in the chloroplast
Lenght	Start position	Length	Start position		
59	94,363	59	94,381	F	*ycf*2 (IR)
49	94,373	49	94,391	F	*ycf*2 (IR)
39	41,060	39	43,284	F	*psa*B (1); *psa*A (2) (LSC)
41	101,726	41	123,418	F	between *rps*12 and *trn*V-GAC (1) (IR); *ndh*A intron (2) (SSC)
36	62,424	36	62,450	F	between *ycf*4 and *cem*A (LSC)
33	101,734	33	123,426	F	between *rps*12 and *trn*V-GAC (1) (IR); *ndh*A intron (2) (SSC)
39	45,344	39	101,728	F	*ycf*3 intron (1) (LSC); between *rps*12 and *trn*V-GAC (2) (IR)
39	45,344	39	123,420	F	*ycf*3 intron (1) (LSC); *ndh*A intron (2) (SSC)
32	94,393	32	94,411	F	*ycf*2 (IR)
34	9,999	34	10,024	F	between *trn*S-CGA and *trn*R-UCU (LSC)
31	14,288	31	14,315	F	between *atp*H and *atp*I (LSC)
31	67,904	31	67,926	F	between *psb*E and *pet*L (LSC)
36	38,652	36	38,679	F	between *psb*Z and *trn*G-GCC (LSC)
34	52,262	34	52,287	F	between *ndh*C and *trn*C-ACA (LSC)
32	91,930	32	91,951	F	*ycf*2 (IR)
32	94,375	32	94,411	F	*ycf*2 (IR)
31	52,307	31	52,328	F	between *ndh*C and *trn*C-ACA (LSC)
31	52,360	31	52,361	F	between *ndh*C and *trn*C-ACA (LSC)
30	45,356	30	101,740	F	*ycf*3 intron (1) (LSC); between *rps*12 and *trn*V-GAC (2) (IR)
56	9,623	56	9,623	P	between *trn*S-CGA and *trn*R-UCU (LSC)
56	30,648	56	30,648	P	between *pet*N and *psb*M (LSC)
52	52,438	52	52,438	P	between *ndh*C and *trn*C-ACA (LSC)
42	76,868	42	76,868	P	between *psb*T and *pbf*1 (LSC)

32	48	32	48	P	before *trn*H-GUG (LSC)
32	30,548	32	30,548	P	between *pet*N and *psb*M (LSC)
38	6,543	38	6,543	P	between *trn*Q-UUG and *psb*K (LSC)
30	7,792	30	46,639	P	*trn*S-GCU (1); *trn*S-GGA (2) (LSC)
32	54,035	32	54,035	P	between *trn*M-CAU and *atp*E (LSC)
30	62,372	30	62,400	P	between *ycf*4 and *cem*A (LSC)
30	72,311	30	72,344	P	between *rps*12 and *clp*P (LSC)
31	8,144	31	82,834	P	between *trn*S-GCU and *trn*S-CGA (1); *rpl*36 and *rps*8 (2) (LSC)
31	32,917	31	32,917	P	between *trn*E-UUC and *trn*T-GGU (LSC)
30	66,180	30	66,230	P	between *pet*A and *psb*J (LSC)
31	31	31	31	R	before *trn*H-GUG (LSC)
33	4,696	33	8,095	R	between *trn*K and *rps*16 (LSC)
33	126,684	33	126,694	R	between *rps*15 and *ycf*1 (SSC)
30	52,357	30	68,680	R	between *ndh*C and *trn*C-ACA (1); between *trn*W-CCA and *trn*P-UGG (2) (LSC)
31	38,642	31	62,424	R	between *psb*Z and *trn*G-GCC (1); between *ycf*4 and *cem*A (LSC)
31	38,642	31	62,450	R	between *psb*Z and *trn*G-GCC (1); between *ycf*4 and *cem*A (2) (LSC)

Clusia panapanari (Aubl.) Choisy					
First repeat		Second repeat		Orientation	Position in the chloroplast
Lenght	Start position	Length	Start position		
41	100,775	41	123,471	F	between *rps*12 and *trn*V-GAC (1) (IR); *ndh*A intron (2) (SSC)
40	115,694	40	115,721	F	between *ndh*F and *rpl*32 (SSC)
39	45,289	39	100,777	F	*ycf*3 intron (1) (LSC); between *rps*12 and *trn*V-GAC (2) (IR)
39	45,289	39	123,473	F	*ycf*3 intron (1) (LSC); *ndh*A intron (2) (SSC)
41	40,993	41	43,221	F	*psa*B (1); *psa*A (2) (LSC)
41	93,362	41	93,380	F	*ycf*2 (IR)
31	93,372	31	93,390	F	*ycf*2 (IR)
32	90,923	32	90,944	F	*ycf*2 (IR)

30	7,574	30	64,218	F	between *trn*Q-UUG and *psb*K (1); between *pet*A and *psb*J (2) (LSC)
43	10,756	43	10,756	P	between *trn*S-CGA and *trn*R-UCU (LSC)
45	75,616	45	75,616	P	between *psb*T and *pbf*1 (LSC)
39	79,365	39	79,365	P	between *pet*D and *rpo*A (LSC)
34	126,769	34	126,769	P	between *rps*15 and *ycf*1 (SSC)
42	4,834	42	68,526	P	between *trn*K and *rps*16 (1); *psa*J and *rpl*33 (2) (LSC)
38	115,256	38	115,256	P	between *ndh*F and *rpl*32 (SSC)
30	96,085	30	96,119	P	between *ycf*15 and *trn*L-CAA (IR)
36	4,847	36	68,519	P	between *trn*K and *rps*16 (1); between *psa*J and *rpl*33 (2) (LSC)
30	8,557	30	46,607	P	*trn*S-GCU (1); *trn*S-GGA (2) (LSC)
35	17,647	35	17,647	P	between *rps*2 and *rpo*C2 (LSC)
32	118,390	32	118,427	P	between *ccs*A and *ndh*D (SSC)
30	111,056	30	111,056	P	between *trn*R-AGC and *trn*N-GUU (IR)
31	71,331	31	71,331	P	between *rps*12 and *clp*P (LSC)
31	84,480	31	84,480	P	between *rpl*16 and *rps*3 (LSC)
31	98,109	31	98,109	P	*ndh*B intron (IR)
30	37,253	30	46,607	P	*trn*S-UGA (1); *trn*S-GGA (2) (LSC)
30	38,435	30	116,033	P	between *psb*Z and *trn*G-GCC (1) (LSC); between *rpl*32 and *trn*L-UAG (2) (SSC)
30	45,290	30	77,028	P	*ycf*3 intron (1); *pet*B intron (2) (LSC)
31	69,876	31	69,876	R	between *rps*16 and *rpl*20 (LSC)

Cratoxylum cochinchinense (Lour.) Blume					
First repeat		Second repeat		Orientation	Position in the chloroplast
Lenght	Start position	Length	Start position		
111	88,106	111	88,203	F	between *trn*I-CAU and *ycf*2 (IR)
87	94,247	87	94,283	F	*ycf*2 (IR)
75	88,110	75	88,334	F	between *trn*I-CAU and *ycf*2 (IR)

75	88,207	75	88,334	F	between *trn*I-CAU and *ycf*2 (IR)
79	40,110	79	42,334	F	*psa*B (1); *psa*A (2) (LSC)
70	94,260	70	94,296	F	*ycf*2 (IR)
57	69,074	57	69,131	F	*rps*18 (LSC)
50	72,716	50	72,742	F	between *clp*P and *psb*B (LSC)
49	88,076	49	88,402	F	between *trn*I-CAU and *ycf*2 (IR)
49	88,124	49	88,432	F	between *trn*I-CAU and *ycf*2 (IR)
49	88,221	49	88,432	F	between *trn*I-CAU and *ycf*2 (IR)
49	88,348	49	88,432	F	between *trn*I-CAU and *ycf*2 (IR)
51	90,205	51	90,226	F	*ycf*2 (IR)
55	68,808	55	68,829	F	*rps*18 (LSC)
44	88,516	44	88,537	F	*ycf*2 (IR)
42	88,282	42	88,393	F	*ycf*2 (IR)
51	94,247	51	94,319	F	*ycf*2 (IR)
39	99,923	39	122,231	F	between *rps*12 and *trn*V-GAC (1) (IR); *ndh*A intron (2) (SSC)
45	90,401	45	90,422	F	*ycf*2 (IR)
41	40,148	41	42,372	F	*psa*B (1); *psa*A (2) (LSC)
41	111,522	41	111,543	F	between *trn*N-GUU and *ndh*F (IR)
37	86,487	37	86,507	F	*rpl*2 intron (IR)
46	58,310	46	58,340	F	*acc*D (LSC)
40	31,026	40	76,324	F	between *psb*M and *trn*D-GUC (1); *pet*B intron (2) (LSC)
40	68,823	40	68,844	F	*rps*18 (LSC)
43	111,471	43	111,483	F	between *trn*N-GUU and *ndh*F (IR)
36	92,852	36	94,382	F	*ycf*2 (IR)
36	92,864	36	94,292	F	*ycf*2 (IR)
44	92,864	44	94,328	F	*ycf*2 (IR)
41	68,808	41	68,850	F	*rps*18 (LSC)
33	88,076	33	88,291	F	between *trn*I-CAU and *ycf*2 (IR)
39	44,723	39	99,923	F	*ycf*3 intron (1) (LSC); between *rps*12 and *trn*V-GAC (2) (IR)
39	44,723	39	122,231	F	*ycf*3 intron (1) (LSC); intron *ndh*A (2) (SSC)

39	94,271	39	94,361	F	*ycf*2 (IR)
39	94,307	39	94,361	F	*ycf*2 (IR)
32	88,185	32	88,393	F	between *trn*I-CAU and *ycf*2 (IR)
32	92,868	32	94,260	F	*ycf*2 (IR)
38	94,260	38	94,332	F	*ycf*2 (IR)
39	94,343	39	94,349	F	*ycf*2 (IR)
30	13,553	30	13,581	F	between *atp*F and *atp*H (LSC)
84	1,706	84	57,034	P	between *psb*A and *trn*K (1); between *rbc*L and *acc*D (2) (LSC)
83	1,617	83	57,122	P	between *psb*A and *trn*K (1); between *rbc*L and *acc*D (2) (LSC)
79	72,924	79	105,889	P	between *clp*P and *psb*B (1) (LSC); between *trn*A and *rrn*23 (2) (IR)
66	1,634	66	57,122	P	between *psb*A and *trn*K (1); between *rbc*L and *acc*D (2) (LSC)
48	72,955	48	105,889	P	between *clp*P and *psb*B (1) (LSC); between *trn*A and *rrn*23 (2) (IR)
47	8,578	47	8,578	P	between *trn*S-GCU and *trn*G-UCC (LSC)
49	78,978	49	78,978	P	between *pet*D and *rpo*A (LSC)
41	71,002	41	71,002	P	between *rps*12 and *clp*P (LSC)
34	125,720	34	125,720	P	between *rps*15 and *ycf*1 (SSC)
36	8,209	36	45,883	P	*trn*S-GCU (1); *trn*S-GGA (2) (LSC)

Garcinia gummi-gutta (L.) N.Robson

First repeat		Second repeat		Orientation	Position in the chloroplast
Lenght	Start position	Length	Start position		
71	92,466	71	92,484	F	*ycf*2 (IR)
59	92,491	59	92,509	F	*ycf*2 (IR)
49	92,501	49	92,519	F	*ycf*2 (IR)
41	99,858	41	121,240	F	between *rps*12 and *trn*V-GAC (1) (IR); *ndh*A intron (2) (SSC)
46	92,473	46	92,509	F	*ycf*2 (IR)
39	44,435	39	99,860	F	*ycf*3 intron (1) (LSC); between *rps*12 and *trn*V-GAC (2) (IR)
39	44,435	39	121,242	F	*ycf*3 intron (1) (LSC); *ndh*A intron (2) (SSC)
41	40,072	41	42,300	F	*psa*B (1); *psa*A (2) (LSC)

43	92,457	43	92,475	F	*ycf*2 (IR)
41	92,491	41	92,527	F	*ycf*2 (IR)
36	92,483	36	92,519	F	*ycf*2 (IR)
32	60,815	32	60,848	F	between *ycf*4 and *cem*A (LSC)
31	10,584	31	10,606	F	between *trn*R-UCU and *atp*A (LSC)
34	10,043	34	10,047	F	between *trn*G-UCC and *trn*R-UCU (LSC)
34	92,466	34	92,520	F	*ycf*2 (IR)
31	92,501	31	92,537	F	*ycf*2 (IR)
33	92,457	33	92,511	F	*ycf*2 (IR)
30	10,045	30	10,051	F	between *trn*G-UCC and *trn*R-UCU (LSC)
30	43,592	30	43,617	F	between *psa*A and *ycf*3 (LSC)
30	45,991	30	45,992	F	between *trn*S-GGA and *rps*4 (LSC)
32	3,791	32	3,817	F	*trn*K intron (LSC)
32	90,028	32	90,049	F	*ycf*2 (IR)
30	1,874	30	6,466	F	*trn*K intron (1); between *rps*16 and *trn*Q-UUG (LSC)
30	10,049	30	10,051	F	between *trn*G-UCC and *trn*R-UCU (LSC)
51	74,764	51	74,764	P	between *psb*T and *psb*N (LSC)
47	47,232	47	47,232	P	between *trn*T-UGU and *trn*L-UAA (LSC)
44	2,882	44	2,882	P	*trn*K intron (LSC)
34	124,577	34	124,577	P	between *rps*15 and *ycf*1 (SSC)
45	78,542	45	78,542	P	between *pet*D and *rpo*A (LSC)
43	100,374	43	100,374	P	between *rps*12 and *trn*V-GAC (IR)
37	28,628	37	95,933	P	between *rpo*B and *trn*C-GCA (1) (LSC); between *trn*L-CAA and *ndh*B + *ndh*B (2) (IR)
30	43,731	30	43,731	P	between *psa*A and *ycf*3 (LSC)
30	7,867	30	45,753	P	*trn*S-GCU (1); *trn*S-GGA (2) (LSC)
35	17,349	35	17,349	P	between *rps*2 and *rpo*C2 (LSC)
32	10,050	32	10,050	P	between *trn*G-UCC and *trn*R-UCU (LSC)
32	109,938	32	109,938	P	between *trn*R-ACG and *trn*N-GUU (IR)

33	81,222	33	81,222	P	between *rps*8 and *rpl*14 (LSC)
30	110,164	30	110,164	P	between *trn*R-ACG and *trn*N-GUU (IR)
30	111,243	30	111,243	P	*ycf*1-frag (IR)
31	9,815	31	9,815	P	between *trn*G-UCC and *trn*R-UCU (LSC)
31	52,647	31	52,647	P	between *trn*M-CAU and *atp*E (LSC)
31	70,481	31	70,481	P	between *rps*12 and *clp*P (LSC)
31	97,202	31	97,202	P	*ndh*B intron (IR)
30	36,209	30	45,753	P	*trn*S-UGA (1); *trn*S-GGA (2) (LSC)
30	44,436	30	76,222	P	*ycf*3 intron (1); *pet*B intron (2) (LSC)
30	10,057	30	10,058	C	between *trn*G-UCC and *trn*R-UCU (LSC)
30	10,048	30	10,051	C	between *trn*G-UCC and *trn*R-UCU (LSC)

Kielmeyera appariciana Saddi					
First repeat		Second repeat		Orientation	Position in the chloroplast
Lenght	Start position	Length	Start position		
138	63,167	138	63,199	F	between *ycf*4 and *cem*A (LSC)
106	63,167	106	63,231	F	between *ycf*4 and *cem*A (LSC)
74	63,167	74	63,263	F	between *ycf*4 and *cem*A (LSC)
59	94,985	59	95,003	F	*ycf*2 (IR)
47	41,567	47	43,791	F	*psa*B (1); *psa*A (2) (LSC)
49	94,995	49	95,013	F	*ycf*2 (IR)
42	63,167	42	63,295	F	between *ycf*4 and *cem*A (LSC)
41	102,345	41	124,232	F	between *rps*12 and *trn*V-GAC (1) (IR); *ndh*A intron (2) (SSC)
39	41,575	39	43,799	F	*psa*B (1); *psa*A (2) (LSC)
46	52,761	46	52,774	F	between *ndh*C and *trn*C-ACA (LSC)
39	45,856	39	102,347	F	*ycf*3 intron (1) (LSC); between *rps*12 and *trn*V-GAC (2) (IR)
39	45,856	39	124,234	F	*ycf*3 intron (1) (LSC); *ndh*A intron (2) (SSC)
32	95,015	32	95,033	F	*ycf*2 (IR)
31	52,780	31	52,793	F	between *ndh*C and *trn*C-ACA (LSC)

32	92,552	32	92,573	F	*ycf*2 (IR)
32	94,997	32	95,033	F	*ycf*2 (IR)
31	39,332	31	39,373	F	between *trn*G-GCC and *trn*M-CAU (LSC)
30	45,868	30	102,359	F	*ycf*3 intron (1) (LSC); between *rps*12 and *trn*V-GAC (2) (IR)
30	59,368	30	59,402	F	between *rbc*L and *acc*D (LSC)
42	77,329	42	77,329	P	between *psb*T and *pbf*1 (LSC)
49	127,547	49	127,547	P	between *rps*15 and *ycf*1 (LSC)
30	8,523	30	47,161	P	between *psb*K and *psb*I (1); *trn*S-GGA (2) (LSC)
36	81,050	36	81,050	P	between *pet*D and *rpo*A (LSC)
32	54,753	32	54,753	P	between *trn*M-CAU and *atp*E (LSC)
33	130,114	33	130,114	P	*ycf*1 (SSC)
31	33,361	31	33,361	P	between *trn*E-UUC and *trn*T-GGU (LSC)
31	38,610	31	38,610	P	between *psb*Z and *trn*G-GCC (LSC)
34	33,837	34	33,837	R	between *trn*E-UUC and *trn*T-GGU (LSC)
33	8,835	33	73,268	R	between *trn*S-GCU and *trn*S-CGA (1); *clp*P intron (2) (LSC)
30	52,762	30	52,794	R	between *ndh*C and *trn*C-ACA (LSC)
30	52,775	30	52,794	R	between *ndh*C and *trn*C-ACA (LSC)

Kielmeyera coriacea Mart. & Zucc.					
First repeat		Second repeat		Orientation	Position in the chloroplast
Lenght	Start position	Length	Start position		
52	84,454	52	84,505	F	between *rps*8 and *rpl*14 (LSC)
59	95,600	59	95,618	F	*ycf*2 (IR)
47	41,913	47	44,137	F	*psa*B (1); *psa*A (2) (LSC)
49	95,610	49	95,628	F	*ycf*2 (IR)
41	102,967	41	124,742	F	between *rps*12 and *trn*V-GAC (1) (IR); *ndh*A intron (2) (SSC)
39	41,921	39	44,145	F	*psa*B (1); *psa*A (2) (LSC)
39	46,211	39	102,969	F	*ycf*3 intron (1) (LSC); between *rps*12 and *trn*V-GAC (2) (IR)

39	46,211	39	124,744	F	*ycf*3 intron (1) (LSC); *ndh*A intron (2) (SSC)
38	29,533	38	29,534	F	between *rpo*B and *trn*C-GCA (LSC)
32	95,630	32	95,648	F	*ycf*2 (IR)
37	29,522	37	29,523	F	between *rpo*B and *trn*C-GCA (LSC)
31	29,500	31	29,501	F	between *rpo*B and *trn*C-GCA (LSC)
36	53,137	36	53,150	F	between *ndh*C and *trn*C-ACA (LSC)
33	39,249	33	39,285	F	between *psb*Z and *trn*G-GCC (LSC)
34	29,498	34	29,537	F	between *rpo*B and *trn*C-GCA (LSC)
31	53,146	31	53,159	F	between *ndh*C and *trn*C-ACA (LSC)
33	29,500	33	29,540	F	between *rpo*B and *trn*C-GCA (LSC)
30	29,500	30	29,502	F	between *rpo*B and *trn*C-GCA (LSC)
32	93,167	32	93,188	F	*ycf*2 (IR)
32	95,612	32	95,648	F	*ycf*2 (IR)
31	29,500	31	29,541	F	between *rpo*B and *trn*C-GCA (LSC)
31	29,501	31	29,539	F	between *rpo*B and *trn*C-GCA (LSC)
31	53,127	31	53,140	F	between *ndh*C and *trn*C-ACA (LSC)
30	38,176	30	38,195	F	between *trn*S-UGA and *psb*Z (LSC)
30	46,223	30	102,981	F	*ycf*3 intron (1) (LSC); between *rps*12 and *trn*V-GAC (2) (IR)
30	59,714	30	59,748	F	between *rbc*L and *acc*D (LSC)
56	31,346	56	31,346	P	between *pet*N and *psb*M (LSC)
42	77,905	42	77,905	P	between *psb*T and *pbf*1 (LSC)
49	128,052	49	128,052	P	between *rps*15 and *ycf*1 (SSC)
40	70,860	40	70,860	P	between *psa*J and *rpl*33 (LSC)
30	8,501	30	47,510	P	*trn*S-GCU (1); *trn*S-GGA (2) (LSC)
36	81,626	36	81,626	P	between *pet*D and *rpo*A (LSC)
35	85,798	35	85,798	P	*rpl*16 intron (LSC)
32	10,820	32	10,820	P	between *trn*S-CGA and *trn*R-UCU (LSC)
32	55,094	32	55,094	P	between *trn*M-CAU and *atp*E (LSC)
31	33,694	31	33,694	P	between *trn*E-UUC and *trt*T-GGU (LSC)

31	38,966	31	38,966	P	between *psb*Z and *trn*G-GCC (LSC)
30	8,810	30	73,850	R	between *trn*S-GCU and *trn*S-CGA (1); *clp*P intron (2) (LSC)
30	10,820	30	45,496	R	between *trn*S-GCU and *trn*S-CGA (1); between *psa*A and *ycf*3 (LSC)
30	53,141	30	53,160	R	between *ndh*C and *trn*C-ACA (LSC)

Mahuera exstipulata Benth.					
First repeat		Second repeat		Orientation	Position in the chloroplast
Lenght	Start position	Length	Start position		
47	41,903	47	44,127	F	*psa*B (1); *psa*A (2) (LSC)
41	102,787	41	124,635	F	between *rps*12 and *trn*V-GAC (1) (IR); *ndh*A intron (2) (SSC)
39	41,911	39	44,135	F	*psa*B (1); *psa*A (2) (LSC)
39	46,206	39	102,789	F	*ycf*3 intron (1) (LSC); between *rps*12 and *trn*V-GAC (2) (IR)
39	46,206	39	124,637	F	*ycf*3 intron (1) (LSC); *ndh*A intron (2) (SSC)
41	95,768	41	95,786	F	*ycf*2 (LSC)
34	104,022	34	104,038	F	between *rps*12 and *trn*V-GAC (IR)
30	63,300	30	63,314	F	between *ycf*4 and *cem*A (LSC)
31	95,778	31	95,796	F	*ycf*2 (LSC)
32	59,536	32	59,587	F	between *rbc*L and *acc*D (LSC)
32	93,335	32	93,356	F	*ycf*2 (LSC)
31	560	31	84,047	F	between *trn*H-GUG and *psb*A (1); *rpl*36 and *rps*8 (2) (LSC)
30	46,218	30	102,801	F	*ycf*3 intron (1) (LSC); between *rps*12 and *trn*V-GAC (2) (IR)
56	31,385	56	31,385	P	between *psb*M and *trn*D-GUC (LSC)
54	127,972	54	127,972	P	between *rps*15 and *ycf*1 (SSC)
52	31,273	52	31,273	P	between *pet*N and *psb*M (LSC)
42	78,094	42	78,094	P	between *psb*T and *pbf*1 (LSC)
43	53,058	43	53,058	P	between *ndh*C and *trn*C-ACA (LSC)
30	8	30	8	P	before *trn*H-GUG (LSC)

30	8,560	30	47,509	P	*trn*S-GCU (1); *trn*S-GGA (2) (LSC)
36	63,405	36	63,405	P	between *ycf*4 and *cem*A (LSC)
36	81,821	36	81,821	P	between *pet*D and *rpo*A (LSC)
30	5,635	30	5,670	P	*rps*16 intron (LSC)
32	54,917	32	54,917	P	between *tnr*M-CAU and *atp*E (LSC)
30	114,141	30	114,141	P	*ycf*1-frag (IR)
31	33,706	31	33,706	P	between *trn*E-UUC and *trn*T-GGU (LSC)
34	34,242	34	34,242	R	between *trn*E-UUC and *trn*T-GGU (LSC)
30	63,354	30	63,370	R	between *ycf*4 and *cem*A (LSC)

Marathrum foeniculaceum Bonpl.

First repeat		Second repeat		Orientation	Position in the chloroplast
Lenght	Start position	Length	Start position		
46	64,445	46	64,466	F	*rps*18 (LSC)
34	21,603	34	21,637	F	between *trn*S-UGA and *psb*C (LSC)
36	87,130	36	109,143	F	between *rps*12 and *trn*V-GAC (1) (IR); *ndh*A intron (2) (SSC)
31	24,562	31	107,327	F	between *psb*D and *trn*T-GGU (1) (LSC); *ndh*G (SSC)
30	7,840	30	7,846	F	between *trn*V-UAC and *ndh*C (LSC)
30	74,881	30	74,900	F	between *rps*11 and *rpl*36 (LSC)
56	69,420	56	69,420	P	between *psb*T and *psb*N (LSC)
39	13,837	39	87,127	P	*ycf*3 intron (1) (LSC); between *rps*12 and *trn*V-GAC (2) (IR)
41	6,649	41	6,649	P	between *atp*E and *trn*M (LSC)
44	102,656	44	102,656	P	between *rpl*32 and *trn*L (SSC)
32	107,549	32	107,549	P	between *ndh*G and *ndh*I (SSC)
41	2,083	41	2,083	P	between *psb*A and *rbc*L (LSC)
36	4,009	36	4,009	P	between *rbc*L and *atp*B (LSC)
36	13,837	36	109,143	P	*ycf*3 intron (1) (LSC); *ndh*A intron (2) (SSC)
30	12,612	30	49,007	P	*trn*S-GGA (1); *trn*S-GCU (2) (LSC)

32	103,144	32	103,144	P	*ccs*A (SSC)
33	14,738	33	14,738	P	between *ycf*3 and *psa*A (LSC)
31	13,845	31	109,140	P	*ycf*3 intron (1) (LSC); *ndh*A intron (2) (SSC)
31	85,015	31	85,015	P	*ndh*B intron (IR)
30	4,312	30	4,333	P	between *rbc*L and *atp*B (LSC)
30	12,614	30	21,409	P	*trn*S-GGA (1); *trn*S-UGA (2) (LSC)
37	7,836	37	7,836	R	between *trn*V-UAC and *ndh*C (LSC)
31	77,858	31	77,858	R	*rpl*16 intron (LSC)
32	10,654	32	28,498	R	*trn*L-UAA intron (1); between *pet*N and *trn*C-GCA (2) (LSC)
32	65,672	32	65,689	R	between *rpl*20 and *rps*12

Mesua ferrea L.					
First repeat		Second repeat		Orientation	Position in the chloroplast
Lenght	Start position	Length	Start position		
56	31,825	56	31,825	P	between petN-psbM (LSC)
59	5,658	59	5,658	P	*rps*16 intron (LSC)
52	78,668	52	78,668	P	between *psb*T and *psb*N (LSC)
42	47,635	42	69,219	P	between *ycf*3 and *trn*S-GGA (1); between *psb*E and *pet*L (2) (LSC)
42	116,398	42	116,398	P	between *trn*N-GUU and *ndh*F (SSC)
41	82,403	41	82,403	P	between *pet*D and *rpo*A (LSC)
44	63,884	44	63,884	P	between *ycf*4 and *cem*A (LSC)
32	31,725	32	31,725	P	between *pet*N and *psb*M (LSC)
30	8,629	30	47,839	P	between *psb*I and *trn*S-GCU (1); *trn*S-GGA (2) (LSC)
35	131,138	35	131,138	P	*ycf*1 (SSC)
32	35,033	32	35,033	P	between *trn*T-GGU and *psb*D (LSC)
32	55,407	32	55,407	P	between *trn*M-CAU and *atp*F (LSC)
30	30,733	30	30,733	P	between *trn*C-GCA and *pet*N (LSC)
30	31,170	30	31,170	P	between *pet*N and *psb*M (LSC)
31	34,092	31	34,092	P	between *trn*E-UUC and *trn*T-GGU (LSC)

31	39,370	31	39,370	P	between *psb*Z and *trn*G-GCC (LSC)
30	39,478	30	63,901	P	between *psb*Z and *trn*G-GCC (1); between *ycf*4 and *cem*A (2) (LSC)
59	96,360	59	96,378	F	*ycf*2 (IR)
49	96,370	49	96,388	F	*ycf*2 (IR)
41	103,749	41	126,526	F	between *rps*12 and *trn*V-GAC (1) (IR); *ndh*A intron (2) (SSC)
39	42,261	39	44,485	F	*psa*B (1); *psa*A (2) (LSC)
44	96,344	44	96,380	F	*ycf*2 (IR)
39	46,519	39	103,751	F	*ycf*3 intron (1) (LSC); between *rps*12 and *trn*V-GAC (2) (IR)
39	46,519	39	126,528	F	*ycf*3 intron (1) (LSC); *ndh*A intron (2) (SSC)
41	96,360	41	96,396	F	*ycf*2 (IR)
31	61,866	31	61,892	F	between *acc*D and *psa*I (LSC)
37	1,908	37	1,925	F	between *psb*A and *trn*K (LSC)
35	11,415	35	63,862	F	between *trn*R-UCU and *atp*A (1); between *ycf*4 and *cem*A (2) (LSC)
34	104,970	34	104,986	F	between *rps*12 and *trn*V-GAC (IR)
31	96,370	31	96,406	F	*ycf*2 (IR)
32	93,927	32	93,948	F	*ycf*2 (IR)
31	14,604	31	14,627	F	between *atp*F and *atp*H (LSC)
31	59,747	31	59,760	F	between *rbc*L and *acc*D (LSC)
30	46,531	30	103,763	F	*ycf*3 intron (1) (LSC); between *rps*12 and *trn*V-GAC (2) (IR)
34	34,614	34	34,614	R	between *trn*E-UUC and *trn*T-GGU (LSC)
30	19	30	119,053	R	before *trn*H-GUG (1) (LSC); between *ndh*F and *rpl*32 (SSC)
30	8,959	30	8,967	R	between *trn*S-GCU and *trn*G-UCC (LSC)

Tristicha trifaria (Bory ex Willd.) Speng.					
First repeat		Second repeat		Orientation	Position in the chloroplast
Lenght	Start position	Length	Start position		
66	91,510	66	91,576	F	*trn*I-GAU intron (IR)
34	3,620	34	3,636	F	between *rbc*L and *atp*B (LSC)

35	63,744	35	63,765	F	*rps*18 (LSC)
33	53,011	33	53,043	F	between *trn*K and *acc*D (LSC)
30	25,447	30	25,472	F	between *psb*D and *trn*T-GGU (LSC)
31	20,326	31	47,278	F	*trn*G-GGC (1); *trnG*-UUC (2) (LSC)
31	21,603	31	48,825	F	*trn*S-UGA (1); *trn*S-GCU (2) (LSC)
31	83,616	31	83,640	F	between *trn*L-CAA and *ndh*B (IR)
51	68,774	51	68,774	P	between *psb*T and *psb*N (LSC)
44	57,743	44	57,743	P	between *cem*A and *pet*A (LSC)
39	13,985	39	87,421	P	*ycf*3 intron (1) (LSC); between *rps*12 and *trn*V-GAC (2) (IR)
41	53,917	41	53,917	P	*acc*D (LSC)
30	102,258	30	102,258	P	*ccs*A (SSC)
31	85,293	31	85,293	P	*ndh*B intron (IR)
30	12,781	30	48,822	P	*trn*S-GGA (1); *trn*S-GCU (2) (LSC)
30	60,131	30	60,177	P	between *psb*E and *pet*L (LSC)
30	12,781	30	21,600	P	*trn*S-GGA (1); *trn*S-UGA (2) (LSC)
32	63,012	32	63,032	P	between *psa*J and *rpl*33 (LSC)
32	64,019	32	64,019	R	*rps*18 (LSC)
30	64,886	30	71,432	R	between *rpl*20 and *rps*12 (1); *pet*D intron (2) (LSC)

Supplementary Table S2. Statistics of the expected number of genetic differences within each protein-coding gene (CDS) and intergenic spacer (IGS) of 12 clusioid plastomes. The columns from left to right indicate: Name (the name of the region), Region (the region in the plastome, i.e., LSC, SSC or IR), Type (if the region is coding – CDS or intergenic – IGS), Sites (the number of sites), Analyzed positions, Segregating sites (S), Conserved sites (CS), Estimated number of mutations (Eta), Number of parsimony informative sites (PIS), Proportion of PIS (calculated as PIS/NetSites × 100), Nucleotide diversity (Pi), and Average number of substitutions per site (ThetaK).

Name	Region	Type	Sites	Analyzed positions (NetSites)	Segregating sites (S)	Conserved sites (CS)	Estimated number of mutations (Eta)	Parsimony informative sites (PIS)	Proportion of PIS (%)	Nucleotide diversity (Pi)	Average number of substitutions per site (ThetaK)
*acc*D	LSC	CDS	2014	1304	783	521	1133	510	39.11	0.2033	265.05
*acc*D-*psa*I	LSC	IGS	1236	239	166	73	235	79	33.05	0.2132	50.95
*atp*A	LSC	CDS	1533	1512	358	1154	437	150	9.92	0.0632	95.51
*atp*A-*atp*F	LSC	CDS	77	49	18	31	23	6	12.24	0.1023	5.01
*atp*B	LSC	CDS	1497	1473	317	1156	400	149	10.12	0.0598	88.15
*atp*B-*rbc*L	LSC	IGS	1267	602	344	258	468	137	22.76	0.1625	97.81
*atp*E	LSC	CDS	414	402	113	289	132	44	10.95	0.0703	28.26
*atp*F	LSC	CDS	648	540	155	385	178	70	12.96	0.0760	41.06
*atp*F-*atp*H	LSC	IGS	699	258	120	138	147	46	17.83	0.1351	34.85
*atp*H	LSC	CDS	246	246	34	212	42	19	7.72	0.0408	10.03
*atp*H-atpI	LSC	IGS	1515	318	173	145	235	81	25.47	0.1591	50.60
*atp*I	LSC	CDS	744	744	162	582	194	67	9.01	0.0574	42.71
*atp*I-*rps*2	LSC	IGS	330	181	87	94	112	35	19.34	0.1278	23.13
*ccs*A	SSC	CDS	981	948	346	602	454	152	16.03	0.1040	98.58

*ccs*A-*ndh*D	SSC	IGS	491	152	108	44	183	69	45.39	0.2695	40.96
*cem*A	LSC	CDS	756	684	240	444	292	105	15.35	0.0960	65.69
*cem*A-*pet*A	LSC	IGS	305	169	91	78	115	49	28.99	0.1598	27.00
*clp*P	LSC	CDS	711	588	294	294	379	153	26.02	0.1449	85.19
*clp*P-*psb*B	LSC	IGS	800	346	187	159	246	75	21.68	0.1470	50.87
*mat*K	LSC	CDS	1662	1500	687	813	900	332	22.13	0.1351	202.58
*ndh*A	SSC	CDS	1122	1074	336	738	427	161	14.99	0.0897	96.32
*ndh*B	IR	CDS	1533	1479	82	1397	86	26	1.76	0.0122	18.05
*ndh*B-*rps*7	IR	IGS	516	301	61	240	63	29	9.63	0.0467	14.06
*ndh*C	LSC	CDS	363	363	79	284	90	28	7.71	0.0512	18.58
*ndh*C-*trn*V	LSC	IGS	1787	170	122	48	168	44	25.88	0.2010	34.17
*ndh*D	SSC	CDS	1536	1461	448	1013	586	215	14.72	0.0901	131.65
*ndh*D-*psa*C	SSC	IGS	191	98	55	43	78	39	39.80	0.2119	20.77
*ndh*E	SSC	CDS	306	303	79	224	96	35	11.55	0.0704	21.35
*ndh*E-*ndh*G	SSC	IGS	316	70	50	20	73	25	35.71	0.2086	14.60
*ndh*F	SSC	CDS	2326	2194	864	1330	1207	435	19.83	0.1202	263.65
*ndh*F-*rpl*32	SSC	IGS	1211	190	132	58	184	62	32.63	0.2326	44.20
*ndh*G	SSC	CDS	573	531	157	374	195	77	14.50	0.0840	44.59
*ndh*G-*ndh*I	SSC	IGS	671	34	13	21	18	8	23.53	0.1176	4.00
*ndh*H	SSC	CDS	1185	693	194	499	240	109	15.73	0.0829	57.44

*ndh*H-*rps*15	SSC	IGS	127	98	28	70	33	12	12.24	0.0833	8.16
*ndh*I	SSC	CDS	606	486	131	355	166	65	13.37	0.0769	37.40
*ndh*I-*ndh*A	SSC	IGS	140	77	47	30	66	22	28.57	0.2001	15.41
*ndh*J	LSC	CDS	480	477	117	360	144	51	10.69	0.0651	31.05
*ndh*J-*ndh*K	LSC	IGS	224	64	42	22	57	21	32.81	0.2007	12.85
*ndh*K	LSC	CDS	807	597	152	445	190	68	11.39	0.0684	40.81
*ndh*K-*ndh*C	LSC	IGS	74	43	19	24	26	6	13.95	0.1169	5.03
*pet*A	LSC	CDS	969	963	224	739	276	89	9.24	0.0633	60.95
*pet*A-*psb*J	LSC	IGS	1530	210	169	41	249	79	37.62	0.2525	53.03
*pet*B	LSC	CDS	648	648	122	526	144	56	8.64	0.0516	33.44
*pet*B-*pet*D	LSC	IGS	1015	171	71	100	93	31	18.13	0.1175	20.09
*pet*D	LSC	IGS	576	480	75	405	90	42	8.75	0.0466	22.37
*pet*D-*rpo*A	LSC	IGS	300	77	55	22	77	29	37.66	0.2179	16.78
*pet*G	LSC	CDS	114	114	20	94	24	7	6.14	0.0440	5.01
*pet*G-*trn*W	LSC	IGS	168	96	47	49	67	27	28.13	0.1837	17.64
*pet*L	LSC	CDS	96	96	33	63	39	13	13.54	0.0891	8.55
*pet*L-*pet*G	LSC	IGS	314	166	91	75	123	37	22.29	0.1545	25.65
*pet*N	LSC	CDS	90	90	10	80	10	3	3.33	0.0225	2.03
*pet*N-*psb*M	LSC	IGS	1421	88	63	25	85	22	25.00	0.1871	16.46
*psa*A	SSC	CDS	2253	2253	375	1878	439	170	7.55	0.0444	99.94

*psa*A-*ycf*3	LSC	IGS	1221	497	272	225	382	124	24.95	0.1628	80.94
*psa*B	LSC	CDS	2205	2205	384	1821	452	181	8.21	0.0471	103.78
*psa*C	LSC	CDS	246	246	42	204	52	17	6.91	0.0476	11.72
*psa*C-*ndh*E	LSC	IGS	350	192	114	78	158	49	25.52	0.1719	33.00
*psa*I	LSC	CDS	114	114	25	89	27	8	7.02	0.0508	5.79
*psa*I-*ycf*4	LSC	IGS	571	189	131	58	195	64	33.86	0.2171	41.03
*psa*J	LSC	CDS	135	135	33	102	43	11	8.15	0.0658	8.88
*psa*J-*rpl*33	LSC	IGS	664	265	180	85	252	78	29.43	0.2037	53.97
*psb*A	LSC	CDS	1089	1062	151	911	169	79	7.44	0.0371	39.40
*psb*A-*trn*K	LSC	IGS	525	60	39	21	61	22	36.67	0.2282	13.69
*psb*B	LSC	CDS	1527	1527	296	1231	380	157	10.28	0.0586	89.42
*psb*B-*psb*T	LSC	IGS	219	83	44	39	64	27	32.53	0.1854	15.38
*psb*C	LSC	CDS	1422	1422	245	1177	307	108	7.59	0.0478	68.00
*psb*C-*trn*S	LSC	IGS	321	173	109	64	154	60	34.68	0.2115	36.59
*psb*D	LSC	CDS	1062	1062	154	908	184	59	5.56	0.0377	40.01
*psb*E	LSC	CDS	252	252	40	212	43	15	5.95	0.0371	9.36
*psb*E-*pet*L	LSC	IGS	1641	326	188	138	249	74	22.70	0.1598	52.09
*psb*F	LSC	CDS	120	120	23	97	26	12	10.00	0.0525	6.29
*psb*H	LSC	CDS	222	222	50	172	56	22	9.91	0.0603	13.40
*psb*H-*pet*B	LSC	IGS	197	108	56	52	76	20	18.52	0.1435	15.50

*psb*I	LSC	CDS	111	111	21	90	24	14	12.61	0.0605	6.72
*psb*I-*trn*S	LSC	IGS	210	86	52	34	72	18	20.93	0.1628	14.00
*psb*J	LSC	CDS	123	123	24	99	27	11	8.94	0.0469	5.77
*psb*J-*psb*L	LSC	IGS	196	110	37	73	46	11	10.00	0.0783	8.62
*psb*K	IR	CDS	186	186	40	146	47	15	8.06	0.0525	9.76
*psb*K-*psb*I	IR	IGS	636	128	71	57	90	36	28.13	0.1551	19.86
*psb*L	LSC	CDS	225	117	12	105	13	3	2.56	0.0226	2.64
*psb*M	LSC	CDS	108	105	16	89	18	5	4.76	0.0365	3.83
*psb*M-*trn*D	LSC	IGS	1533	292	136	156	179	73	25.00	0.1437	41.97
*psb*N	LSC	CDS	132	132	15	117	17	3	2.27	0.0239	3.15
*psb*N-*psb*H	LSC	IGS	150	103	31	72	39	9	8.74	0.0705	7.26
*psb*T	LSC	CDS	108	102	13	89	16	7	6.86	0.0392	4.00
*psb*T-*psb*N	LSC	IGS	93	59	29	30	42	13	22.03	0.1460	8.62
*psb*Z	LSC	CDS	189	189	41	148	47	19	10.05	0.0569	10.76
*psb*Z-*trn*G	LSC	IGS	1201	86	61	25	88	29	33.72	0.2184	18.78
*rbc*L	LSC	CDS	1464	1428	277	1151	328	140	9.80	0.0538	76.83
*rbc*L-*acc*D	LSC	IGS	1395	327	223	104	311	113	34.56	0.2083	68.12
*rpl*14	LSC	CDS	369	369	103	266	119	45	12.20	0.0748	27.62
*rpl*14-*rpl*16	LSC	IGS	172	73	40	33	55	18	24.66	0.1653	12.06
*rpl*16	LSC	CDS	435	408	117	291	151	57	13.97	0.0849	34.65

*rpl*16-*rps*3	LSC	IGS	225	46	24	22	38	11	23.91	0.1826	8.40
*rpl*2	IR	CDS	873	825	77	748	82	28	3.39	0.0202	16.69
*rpl*20	LSC	CDS	366	348	131	217	156	46	13.22	0.0960	33.41
*rpl*20-*rps*12	LSC	IGS	972	690	336	354	435	134	19.42	0.1331	91.85
*rpl*22	LSC	CDS	441	360	164	196	209	92	25.56	0.1395	50.22
*rpl*22-*rps*19	LSC	IGS	192	21	18	3	28	11	52.38	0.3248	6.82
*rpl*23	IR	CDS	282	282	11	271	11	0	0.00	0.0071	2.00
*rpl*23-*trn*M	IR	IGS	173	157	31	126	37	17	10.83	0.0534	8.38
*rpl*32	SSC	CDS	238	76	17	59	17	9	11.84	0.0651	4.95
*rpl*32-*trn*L	SSC	IGS	2175	68	39	29	52	23	33.82	0.1768	12.03
*rpl*33	LSC	CDS	207	198	64	134	86	35	17.68	0.0991	19.63
*rpl*33-*rps*18	LSC	IGS	265	143	94	49	138	40	27.97	0.1938	27.72
*rpl*36	LSC	CDS	114	114	28	86	30	13	11.40	0.0617	7.04
*rpl*36-*rps*8	LSC	IGS	591	71	46	25	72	23	32.39	0.2214	15.72
*rpo*A	LSC	CDS	1050	990	312	678	393	138	13.94	0.0869	86.08
*rpo*A-*rps*11	LSC	IGS	114	61	26	35	32	11	18.03	0.1164	7.10
*rpo*B	LSC	CDS	3216	3213	765	2448	918	335	10.43	0.0633	203.46
*rpo*B-*trn*C	LSC	IGS	1724	341	193	148	261	71	20.82	0.1532	52.23
*rpo*C1	LSC	CDS	2108	2022	483	1539	582	214	10.58	0.0644	130.22
*rpo*C2	LSC	CDS	4318	4040	1394	2646	1715	595	14.73	0.0926	374.03

*rpo*C2-*rpo*C1	LSC	IGS	221	149	79	70	104	37	24.83	0.1493	22.24
*rps*11	LSC	CDS	426	417	143	274	172	67	16.07	0.0950	39.62
*rps*11-*rpl*36	LSC	IGS	204	80	54	26	71	27	33.75	0.1873	14.99
*rps*12	IR	CDS	381	372	47	325	55	19	5.11	0.0328	12.21
*rps*12-*clp*P	LSC	IGS	347	117	73	44	106	32	27.35	0.1904	22.28
*rps*14	LSC	CDS	303	303	74	229	89	39	12.87	0.0704	21.33
*rps*14-*psa*B	LSC	IGS	199	49	39	10	50	23	46.94	0.2268	11.12
*rps*15	SSC	CDS	282	221	73	148	88	34	15.38	0.0933	20.63
*rps*16	LSC	CDS	293	63	15	48	18	4	6.35	0.0899	5.67
*rps*16-*trn*Q	LSC	IGS	1241	82	35	47	37	6	7.32	0.1246	10.21
*rps*18	LSC	CDS	442	293	75	218	90	32	10.92	0.0654	19.17
*rps*18-*rpl*20	LSC	IGS	365	173	121	52	172	55	31.79	0.2080	35.99
*rps*19	IR	CDS	282	279	68	211	75	29	10.39	0.0619	17.26
*rps*19-*rpl*2	IR	IGS	95	57	18	39	20	9	15.79	0.0830	4.73
*rps*2	LSC	CDS	714	711	180	531	210	75	10.55	0.0659	46.85
*rps*2-*rpo*C2	LSC	IGS	451	163	94	69	122	45	27.61	0.1664	27.12
*rps*3	LSC	CDS	675	657	245	412	306	120	18.26	0.1065	70.00
*rps*3-*rpl*22	LSC	IGS	161	41	23	18	34	12	29.27	0.1873	7.68
*rps*4	LSC	CDS	606	606	174	432	205	65	10.73	0.0725	43.92
*rps*4-*trn*T	LSC	IGS	585	152	82	70	102	36	23.68	0.1465	22.27

*rps*7	IR	CDS	473	361	20	341	20	5	1.39	0.0118	4.26
*rps*7-*rps*12	IR	IGS	69	44	10	34	12	2	4.55	0.0487	2.14
*rps*8	LSC	CDS	411	405	140	265	171	63	15.56	0.0956	38.73
*rps*8-*rpl*14	LSC	IGS	599	148	115	33	172	58	39.19	0.2467	36.51
*rrn*16	IR	CDS	1491	1489	46	1443	47	19	1.28	0.0073	10.86
*rrn*16-*trn*E	IR	IGS	314	277	58	219	62	24	8.66	0.0532	14.73
*rrn*23	IR	CDS	2812	2804	83	2721	89	33	1.18	0.0071	19.77
*rrn*23-*rrn*4.5	IR	IGS	100	96	14	82	16	5	5.21	0.0330	3.17
*rrn*4.5	IR	CDS	103	103	4	99	4	1	0.97	0.0072	0.74
*rrn*4.5-*rrn*5	IR	IGS	248	206	50	156	55	13	6.31	0.0520	10.71
*rrn*5	IR	CDS	121	121	4	117	4	3	2.48	0.0097	1.18
*rrn*5-*trn*R	IR	IGS	280	206	52	154	60	27	13.11	0.0660	13.59
*trn*A-*rrn*23	IR	IGS	166	152	27	125	28	12	7.89	0.0425	6.46
*trn*C-*pet*N	LSC	IGS	1293	228	135	93	198	71	31.14	0.1879	42.83
*trn*D-*trn*Y	LSC	IGS	626	142	97	45	133	39	27.46	0.1919	27.24
*trn*E-*trn*A	IR	IGS	83	58	15	43	15	7	12.07	0.0584	3.38
*trn*E-*trn*T	LSC	IGS	1016	135	99	36	141	45	33.33	0.2217	29.94
*trn*F-*ndh*J	LSC	IGS	861	108	77	31	117	34	31.48	0.2160	23.33
*trn*G-*trn*M	LSC	IGS	247	15	10	5	16	7	46.67	0.2709	4.06

*trn*G-*trn*R	LSC	IGS	1123	50	32	18	50	19	38.00	0.2400	12.00
*trn*H-*psb*A	LSC	IGS	622	81	50	31	64	30	37.04	0.1978	16.03
*trn*I-*ycf*2	IR	IGS	536	88	18	70	19	4	4.55	0.0398	3.50
*trn*K-*rps*16	LSC	IGS	820	384	100	284	109	57	14.84	0.0978	37.57
*trn*K-*trn*Q	LSC	IGS	1667	242	148	94	218	56	23.14	0.1818	44.00
*trn*L-*ccs*A	SSC	IGS	176	47	33	14	40	17	36.17	0.2100	9.87
*trn*L-*ndh*B	IR	IGS	640	412	82	330	86	40	9.71	0.0493	20.31
*trn*L-*trn*F	LSC	IGS	525	28	19	9	26	9	32.14	0.2019	5.65
*trn*M-*atp*E	LSC	IGS	386	108	74	34	105	27	25.00	0.1898	20.50
*trn*M-*rps*14	LSC	IGS	175	65	45	20	60	30	46.15	0.2140	13.91
*trn*N-*ndh*F	IR	IGS	2878	924	456	468	562	136	14.72	0.1161	107.28
*trn*P-*psa*J	LSC	IGS	661	303	190	113	272	96	31.68	0.1953	59.18
*trn*R-*atp*A	LSC	IGS	455	51	38	13	53	21	41.18	0.2423	12.36
*trn*R-*trn*N	IR	IGS	751	354	82	272	90	24	6.78	0.0514	18.19
*trn*S-*psb*Z	LSC	IGS	591	269	181	88	257	89	33.09	0.2054	55.24
*trn*S-*rps*4	LSC	IGS	485	209	161	48	220	66	31.58	0.2171	45.37
*trn*S-*trn*G	LSC	IGS	1594	179	119	60	162	64	35.75	0.2124	38.03
*trn*T-*psb*D	LSC	IGS	1932	706	425	281	580	202	28.61	0.1793	126.59

*trn*T-*trn*L	LSC	IGS	644	230	142	88	199	58	25.22	0.1765	40.59
*trn*V-*rps*12	IR	IGS	2090	877	176	701	180	73	8.32	0.0452	39.67
*trn*V-*rrn*16	IR	IGS	239	224	32	192	36	12	5.36	0.0330	7.40
*trn*V-*trn*M	LSC	IGS	278	147	118	29	163	45	30.61	0.2228	32.76
*trn*W-*trn*P	LSC	IGS	350	114	71	43	102	32	28.07	0.1890	21.55
*trn*Y-*trn*E	LSC	IGS	65	44	28	16	35	5	11.36	0.1451	6.38
*ycf*1	IR/SSC	CDS	5318	224	131	93	163	34	15.18	0.1471	32.95
*ycf*15-*trn*L	IR	IGS	791	577	79	498	82	18	3.12	0.0328	18.95
*ycf*1-*rps*15	SSC	IGS	1243	182	139	43	202	66	36.26	0.2444	44.49
*ycf*2	IR	CDS	7413	112	27	85	32	10	8.93	0.0564	6.32
*ycf*2-*ycf*15	IR	IGS	986	127	17	110	19	6	4.72	0.0382	4.85
*ycf*3	LSC	CDS	516	501	78	423	92	31	6.19	0.0407	20.37
*ycf*3-*trn*S	LSC	IGS	604	202	126	76	172	47	23.27	0.1708	34.50
*ycf*4	LSC	CDS	642	555	165	390	197	64	11.53	0.0780	43.28
*ycf*4-*cem*A	LSC	IGS	1536	108	75	33	110	33	30.56	0.2100	22.68

Supplementary Table S3. Voucher information from newly sequenced specimens.

Species	Collector, collector number and herbarium	Identification	Collection date	Locality	Geographic coordinates
Calophyllum brasiliense Cambess.	N. Hind 4260, UEC	M. C. E. Amaral & V. Bittrich[1]	14/Nov/1996	Rio de Contas, Brazil	-
Caraipa heterocarpa Ducke	F. N. Cabral FC705, UEC	F. N. Cabral	14/Mar/2013	Manaus, Brazil	2°57'11.4"S 59°57'41.4"W
Kielmeyera appariciana Saddi	R. J. Trad 192, UEC	R. J. Trad	01/Dec/2014	Santana do Riacho, Brazil	19°06'53.0"S 43°41'57.0"W
Kielmeyera coriacea Mart & Zucc.	R. J. Trad 401, UEC	R. J. Trad	08/May/2014	Ipameri, Brazil	16°58'55.0"S 47°44'50.0"W
Mahurea exstipulata Benth.	F.N. Cabral FC1140, UEC	F. N. Cabral	16/Apr/2014	Amajari, Brazil	3°39'08.0"N 61°22'14.2"W

[1]The identification was confirmed by M.C.E. Amaral and V. Bittrich in this case.

Supplementary Material

Title: Calophyllaceae plastomes, their structure and insights in relationships within the clusioids

Authors: Rafaela Jorge Trad*, Fernanda Nunes Cabral, Volker Bittrich, Saura Rodrigues da Silva and Maria do Carmo Estanislau do Amaral

*corresponding author: rafajt@hotmail.com

Supplementary Material S1. Species names and GenBank access number for the sequences included in this study.

Anthodiscus peruanus Baill. - Caryocaraceae - JX661767, JX661840, JX661927, JX661968, JX662049, JX662091, JX662132, JX662178, JX662263, JX662305, JX662344, JX662604, JX662649, JX662693, JX662736, JX662780, JX662827, JX662936, JX662982, JX663016, JX663088, JX663130, JX663178, JX663263, JX663306, JX663350, JX663394, JX663459, JX663535, JX663564, JX663611, JX663658, JX663705, JX663748, JX663788, JX663896, JX663944, JX664031, JX664190, JX664236, JX664281, JX664326, JX664367, JX664516, JX664562, JX664607, JX664680, JX664707, JX664755, JX664835, JX664878, JX664920, JX664985

Averrhoa carambola L. - Oxalidaceae - NC_033350

Azara serrata Ruiz & Pav. - Salicaceae - MH719101

Balanops pachyphylla Baill. ex Guillaumin - Balanopaceae - JX661768, JX661842, JX661887, JX661929, JX661970, JX662006, JX662051, JX662093, JX662134, JX662180, JX662224, JX662265, JX662307, JX662346, JX662488, JX662528, JX662606, JX662651, JX662695, JX662738, JX662782, JX662829, JX662865, JX662938, JX662983, JX663018, JX663090, JX663132, JX663180, JX663265, JX663308, JX663352, JX663396, JX663461, JX663537, JX663566, JX663613, JX663660, JX663707, JX663750, JX663790, JX663898, JX663946, JX663992, JX664033, JX664078, JX664151, JX664192, JX664238, JX664283, JX664328, JX664369, JX664518, JX664564, JX664609, JX664682, JX664709, JX664757, JX664801, JX664837, JX664880, JX664922, JX664987

Bergia texana (Hook.) Seub. - Elatinaceae - JX661769, JX661802, JX661843, JX661930, JX661971, JX662052, JX662094, JX662135, JX662181, JX662225, JX662266, JX662529, JX662607, JX662652, JX662696, JX662739, JX662783, JX662866, JX662939, JX663091, JX663133, JX663181, JX663224, JX663266, JX663397, JX663462, JX663538, JX663567, JX663614, JX663661, JX663708, JX663791, JX663863, JX663899, JX663947, JX663993, JX664034, JX664079, JX664193, JX664284, JX664329, JX664370, JX664446, JX664519, JX664565, JX664610, JX664683, JX664710, JX664838, JX664881, JX664923, JX664988

Bhesa sp. - Centroplacaceae - JX661770, JX661803, JX661844, JX661888, JX661931, JX661972, JX662007, JX662053, JX662095, JX662136, JX662182, JX662226, JX662267, JX662308, JX662347, JX662386, JX662423, JX662456, JX662489, JX662530, JX662569, JX662608, JX662653, JX662697, JX662740, JX662784, JX662830, JX662867, JX662902, JX662940, JX662984, JX663019, JX663061, JX663092, JX663134, JX663182, JX663225, JX663267, JX663309, JX663353, JX663398, JX663436, JX663463, JX663506, JX663539, JX663568, JX663615, JX663662, JX663709, JX663751, JX663792, JX663830, JX663864, JX663900, JX663948, JX663994, JX664035, JX664080, JX664116, JX664152, JX664194,

JX664239, JX664285, JX664330, JX664371, JX664411, JX664447, JX664484, JX664520, JX664566, JX664611, JX664649, JX664684, JX664711, JX664758, JX664802, JX664839, JX664882, JX664924, JX664969, JX664989

Byrsonima crassifolia (L.) Kunth - Malpighiaceae - JX661771, JX661804, JX661845, JX661889, JX661932, JX662096, JX662137, JX662183, JX662309, JX662348, JX662387, JX662609, JX662654, JX662741, JX662785, JX662831, JX662903, JX662941, JX663135, JX663183, JX663268, JX663464, JX663569, JX663616, JX663663, JX663710, JX663752, JX663793, JX663901, JX663949, JX664036, JX664195, JX664372, JX664412, JX664448, JX664521, JX664567, JX664712, JX664925, JX664970, JX664990

Caloncoba echinata (Oliv.) Gilg - Achariaceae - JX661772, JX661805, JX661846, JX661890, JX661933, JX661973, JX662008, JX662054, JX662097, JX662138, JX662184, JX662227, JX662268, JX662310, JX662349, JX662388, JX662424, JX662457, JX662490, JX662531, JX662570, JX662610, JX662655, JX662698, JX662742, JX662786, JX662832, JX662868, JX662904, JX662942, JX662985, JX663020, JX663062, JX663093, JX663136, JX663184, JX663226, JX663269, JX663310, JX663354, JX663399, JX663437, JX663465, JX663507, JX663540, JX663570, JX663617, JX663664, JX663711, JX663753, JX663794, JX663831, JX663865, JX663902, JX663950, JX663995, JX664037, JX664081, JX664117, JX664153, JX664196, JX664240, JX664286, JX664331, JX664373, JX664413, JX664449, JX664485, JX664522, JX664568, JX664612, JX664650, JX664685, JX664713, JX664759, JX664803, JX664840, JX664883, JX664926, JX664991

Casearia nitida Jacq. - Salicaceae - JX661806, JX661847, JX661891, JX661934, JX661974, JX662009, JX662055, JX662098, JX662139, JX662185, JX662228, JX662269, JX662311, JX662350, JX662389, JX662425, JX662458, JX662491, JX662532, JX662571, JX662611, JX662699, JX662743, JX662787, JX662833, JX662869, JX662905, JX662943, JX662986, JX663021, JX663063, JX663094, JX663137, JX663185, JX663227, JX663270, JX663311, JX663355, JX663400, JX663466, JX663508, JX663571, JX663618, JX663665, JX663712, JX663754, JX663795, JX663832, JX663866, JX663903, JX663951, JX663996, JX664038, JX664082, JX664118, JX664154, JX664197, JX664241, JX664287, JX664332, JX664374, JX664414, JX664450, JX664486, JX664523, JX664569, JX664613, JX664651, JX664686, JX664714, JX664760, JX664804, JX664841, JX664884, JX664927, JX664992

Centroplacus glaucinus Pierre - Centroplacaceae - JX661773, JX661848, JX661892, JX662010, JX662099, JX662140, JX662186, JX662229, JX662351, JX662390, JX662492, JX662612, JX662656, JX662700, JX662744, JX662788, JX662834, JX662906, JX662944, JX662987, JX663022, JX663138, JX663186, JX663228, JX663312, JX663356, JX663438, JX663467, JX663541, JX663572, JX663619, JX663666, JX663713, JX663755, JX663796, JX663904, JX663952, JX663997, JX664039, JX664083, JX664155, JX664198, JX664242, JX664375, JX664415, JX664524, JX664570, JX664715, JX664761, JX664805, JX664842, JX664885, JX664928, JX664971, JX664993

Chrysobalanus icaco L. - Chrysobalanaceae - JX661807, JX661849, JX661893, JX661935, JX661975, JX662011, JX662056, JX662100, JX662141, JX662187, JX662230, JX662270, JX662312, JX662352, JX662391, JX662426, JX662459, JX662493, JX662533, JX662572, JX662613, JX662657, JX662701, JX662745, JX662789, JX662870, JX662907, JX662945, JX663023, JX663064, JX663095, JX663139, JX663187, JX663229, JX663271, JX663313, JX663357, JX663401, JX663439, JX663509, JX663573, JX663620, JX663667, JX663714, JX663756, JX663797, JX663833, JX663867, JX663905, JX663953, JX663998, JX664040, JX664084, JX664119, JX664156, JX664199, JX664243, JX664288, JX664333, JX664376, JX664416, JX664451, JX664487, JX664525, JX664571, JX664614, JX664652, JX664687, JX664716, JX664762, JX664806, JX664843, JX664886, JX664929, JX664994

Clusia rosea Jacq. - Clusiaceae - JX661774, JX661808, JX661850, JX661894, JX661936, JX661976, JX662012, JX662057, JX662101, JX662142, JX662188, JX662231, JX662271, JX662313, JX662353, JX662392, JX662427, JX662460, JX662494, JX662534, JX662573, JX662614, JX662658, JX662702, JX662746, JX662790, JX662835, JX662871, JX662908, JX662946, JX662988, JX663024, JX663065, JX663096, JX663140, JX663188, JX663230, JX663272, JX663314, JX663358, JX663402, JX663440, JX663468, JX663510, JX663542, JX663574, JX663621, JX663668, JX663715, JX663757, JX663834, JX663868, JX663906, JX663954, JX663999, JX664041, JX664085, JX664120, JX664157, JX664200, JX664244, JX664289, JX664334, JX664377, JX664417, JX664452, JX664488, JX664526, JX664572, JX664615, JX664653, JX664688, JX664717, JX664763, JX664807, JX664844, JX664887, JX664930, JX664995

Ctenolophon englerianus Mildbr. - Ctenolophonaceae - JX661775, JX661809, JX661851, JX661895, JX662013, JX662102, JX662143, JX662189, JX662535, JX662615, JX662659, JX662703, JX662791, JX662872, JX662947, JX662989, JX663025, JX663141, JX663189, JX663315, JX663359, JX663469, JX663575, JX663622, JX663669, JX663907, JX663955, JX664201, JX664245, JX664290, JX664378, JX664527, JX664573, JX664718, JX664764, JX664845, JX664888, JX664931, JX664996

Dichapetalum zenkeri Engl. - Dichapetalaceae - JX661776, JX661852, JX661896, JX661937, JX662058, JX662103, JX662144, JX662190, JX662272, JX662314, JX662428, JX662461, JX662495, JX662536, JX662574, JX662616, JX662660, JX662704, JX662792, JX662873, JX662948, JX663066, JX663097, JX663142, JX663190, JX663231, JX663273, JX663403, JX663470, JX663576, JX663623, JX663670, JX663798, JX663835, JX663908, JX663956, JX664000, JX664042, JX664121, JX664158, JX664202, JX664291, JX664335, JX664379, JX664489, JX664528, JX664574, JX664616, JX664719, JX664846, JX664889, JX664932, JX664972, JX664997

Elaeodendron orientale Jacq. - Celastraceae - JX661777, JX661810, JX661853, JX661897, JX661938, JX662014, JX662059, JX662104, JX662145, JX662191, JX662232, JX662273, JX662315, JX662354, JX662393, JX662429, JX662462, JX662496, JX662537, JX662575, JX662617, JX662661, JX662705, JX662747, JX662793, JX662836, JX662874, JX662909, JX662949, JX662990, JX663026, JX663067, JX663098, JX663143, JX663191, JX663232, JX663274, JX663316, JX663360, JX663404, JX663441, JX663471, JX663511, JX663543, JX663577, JX663624, JX663671, JX663716, JX663758, JX663799, JX663836, JX663869, JX663909, JX663957, JX664001, JX664043, JX664086, JX664122, JX664159, JX664203, JX664246, JX664292, JX664380, JX664418, JX664453, JX664490, JX664529, JX664575, JX664617, JX664654, JX664689, JX664720, JX664765, JX664808, JX664847, JX664890, JX664933, JX664973, JX664998

Erythroxylum areolatum L. - Erythroxylaceae - JX661811, JX661854, JX661898, JX661939, JX662015, JX662060, JX662105, JX662146, JX662192, JX662233, JX662274, JX662316, JX662355, JX662394, JX662430, JX662463, JX662497, JX662538, JX662576, JX662618, JX662662, JX662706, JX662748, JX662794, JX662837, JX662875, JX662910, JX662950, JX662991, JX663027, JX663068, JX663099, JX663144, JX663192, JX663233, JX663275, JX663317, JX663361, JX663405, JX663442, JX663472, JX663512, JX663578, JX663625, JX663672, JX663717, JX663759, JX663800, JX663837, JX663870, JX663910, JX663958, JX664002, JX664044, JX664087, JX664123, JX664160, JX664204, JX664247, JX664293, JX664336, JX664381, JX664419, JX664454, JX664491, JX664530, JX664576, JX664618, JX664655, JX664690, JX664721, JX664766, JX664809, JX664848, JX664891, JX664934, JX664999

Euphorbia maculata L. - Euphorbiaceae - JX661812, JX661855, JX661899, JX661940, JX661977, JX662016, JX662061, JX662106, JX662147, JX662193, JX662234, JX662275, JX662317, JX662356, JX662395, JX662431, JX662464, JX662498, JX662539, JX662577, JX662619, JX662663, JX662707, JX662749, JX662795, JX662838, JX662876, JX662911, JX662951, JX662992, JX663028, JX663069, JX663100, JX663145, JX663193, JX663234, JX663276, JX663318, JX663362, JX663406, JX663473, JX663513, JX663579, JX663626, JX663673, JX663718, JX663760, JX663801, JX663838, JX663871, JX663911, JX663959, JX664003, JX664045, JX664088, JX664124, JX664161, JX664205, JX664248, JX664294, JX664337, JX664382, JX664420, JX664455, JX664492, JX664531, JX664577, JX664619, JX664656, JX664691, JX664722, JX664767, JX664810, JX664849, JX664892, JX664935, JX665000

Garcinia mangostana L. - Clusiaceae - JX661816, JX661859, JX661902, JX661944, JX661980, JX662020, JX662065, JX662109, JX662151, JX662196, JX662237, JX662279, JX662320, JX662359, JX662399, JX662434, JX662467, JX662502, JX662543, JX662580, JX662622, JX662666, JX662710, JX662752, JX662799, JX662841, JX662880, JX662914, JX662955, JX662996, JX663032, JX663071, JX663104, JX663149, JX663196, JX663237, JX663280, JX663322, JX663365, JX663410, JX663583, JX663630, JX663677, JX663721, JX663763, JX663804, JX663841, JX663874, JX663915, JX663962, JX664006, JX664049, JX664091, JX664127, JX664165, JX664209, JX664252, JX664297, JX664341, JX664385, JX664458, JX664495, JX664535, JX664580, JX664623, JX664659, JX664694, JX664726, JX664771, JX664812, JX664852, JX664895, JX664939, JX665004

Goupia glabra Aubl. - Goupiaceae - JX661781, JX661903, JX662021, JX662110, JX662238, JX662667, JX662800, JX662997, JX663150, JX663238, JX663366, JX663477, JX663631, JX663678, JX663916, JX663963, JX664253, JX664298, JX664386, JX664727, JX664772, JX664896, JX664940, JX664975, JX665005

Humiria balsamifera (Aubl.) J.St.-Hil. - Humiriaceae - JX661782, JX661904, JX661945, JX662022, JX662111, JX662152, JX662360, JX662668, JX662711, JX662753, JX662801, JX662842, JX662881, JX662998, JX663033, JX663151, JX663239, JX663281, JX663323, JX663367, JX663444, JX663478, JX663584, JX663632, JX663679, JX663764, JX663805, JX663917, JX663964, JX664050, JX664254, JX664299, JX664387, JX664536, JX664728, JX664773, JX664853, JX664897, JX664941, JX664976, JX665006

Hypericum fraseri Steud. - Hypericaceae - JX661817, JX661860, JX661905, JX661981, JX662023, JX662066, JX662153, JX662197, JX662239, JX662280, JX662321, JX662361, JX662400, JX662435, JX662468, JX662503, JX662544, JX662581, JX662623, JX662669, JX662712, JX662754, JX662802, JX662843, JX662915, JX662956, JX663034, JX663072, JX663105, JX663152, JX663197, JX663240, JX663282, JX663324, JX663368, JX663411, JX663479, JX663516, JX663585, JX663633, JX663680, JX663722, JX663765, JX663842, JX663875, JX663918, JX663965, JX664007, JX664051, JX664092, JX664128, JX664166, JX664210, JX664255, JX664300, JX664342, JX664388, JX664423, JX664459, JX664496, JX664537, JX664581, JX664624, JX664660, JX664729, JX664774, JX664813, JX664854, JX664898, JX664942, JX665007

Hypericum kalmianum L. - Hypericaceae - JX661783, JX661818, JX661861, JX661906, JX661946, JX661982, JX662024, JX662067, JX662112, JX662154, JX662198, JX662240, JX662281, JX662322, JX662362, JX662401, JX662436, JX662469, JX662504, JX662545, JX662582, JX662624, JX662713, JX662755, JX662803, JX662844, JX662882, JX662916, JX662957, JX662999, JX663035, JX663073, JX663106, JX663153, JX663198, JX663241, JX663283, JX663325, JX663369, JX663412, JX663480, JX663517, JX663547, JX663586, JX663634, JX663681, JX663723, JX663766, JX663843, JX663876, JX663919, JX663966,

JX664008, JX664052, JX664093, JX664129, JX664167, JX664211, JX664256, JX664301, JX664343, JX664389, JX664424, JX664460, JX664497, JX664538, JX664582, JX664625, JX664661, JX664730, JX664775, JX664814, JX664855, JX664943, JX665008

Hypericum perforatum L. - Hypericaceae - JX661784, JX661819, JX661862, JX661907, JX661947, JX661983, JX662025, JX662068, JX662113, JX662155, JX662199, JX662241, JX662282, JX662323, JX662363, JX662402, JX662437, JX662470, JX662505, JX662546, JX662583, JX662625, JX662670, JX662714, JX662756, JX662804, JX662845, JX662917, JX662958, JX663036, JX663107, JX663154, JX663199, JX663242, JX663284, JX663326, JX663370, JX663413, JX663481, JX663518, JX663548, JX663587, JX663635, JX663682, JX663724, JX663767, JX663806, JX663844, JX663877, JX663920, JX663967, JX664009, JX664053, JX664094, JX664130, JX664168, JX664212, JX664257, JX664302, JX664344, JX664425, JX664461, JX664498, JX664539, JX664583, JX664626, JX664662, JX664731, JX664776, JX664815, JX664856, JX664899, JX664944, JX665009

Irvingia malayana Oliv. ex Benn. - Irvingiaceae - JX661785, JX661820, JX661863, JX661908, JX661948, JX661984, JX662026, JX662069, JX662114, JX662156, JX662200, JX662242, JX662283, JX662324, JX662364, JX662403, JX662438, JX662471, JX662506, JX662547, JX662584, JX662626, JX662671, JX662715, JX662757, JX662805, JX662846, JX662883, JX662918, JX662959, JX663037, JX663074, JX663108, JX663155, JX663200, JX663243, JX663285, JX663327, JX663371, JX663414, JX663445, JX663482, JX663519, JX663549, JX663588, JX663636, JX663683, JX663725, JX663768, JX663807, JX663845, JX663878, JX663921, JX663968, JX664010, JX664054, JX664095, JX664131, JX664169, JX664213, JX664258, JX664303, JX664345, JX664390, JX664426, JX664462, JX664499, JX664540, JX664584, JX664627, JX664663, JX664695, JX664732, JX664777, JX664816, JX664857, JX664900, JX664945, JX665010

Ixonanthes sp. - Ixonanthaceae - JX661821, JX661864, JX661909, JX661949, JX661985, JX662027, JX662070, JX662115, JX662157, JX662201, JX662243, JX662284, JX662325, JX662365, JX662404, JX662439, JX662472, JX662507, JX662548, JX662585, JX662627, JX662672, JX662716, JX662758, JX662806, JX662847, JX662884, JX662919, JX662960, JX663000, JX663038, JX663075, JX663109, JX663156, JX663201, JX663286, JX663328, JX663372, JX663415, JX663483, JX663520, JX663550, JX663589, JX663637, JX663684, JX663726, JX663769, JX663808, JX663846, JX663879, JX663922, JX663969, JX664011, JX664055, JX664096, JX664132, JX664170, JX664214, JX664259, JX664304, JX664346, JX664391, JX664427, JX664463, JX664500, JX664541, JX664585, JX664628, JX664664, JX664696, JX664733, JX664778, JX664817, JX664858, JX664901, JX664946, JX665011

Lacistema robustum Schnizl. - Lacistemataceae - JX661786, JX661822, JX661865, JX661910, JX661950, JX661986, JX662028, JX662071, JX662116, JX662158, JX662202, JX662244, JX662285, JX662326, JX662366, JX662405, JX662440, JX662473, JX662508, JX662549, JX662586, JX662628, JX662673, JX662717, JX662759, JX662807, JX662885, JX662920, JX662961, JX663001, JX663039, JX663076, JX663110, JX663157, JX663202, JX663244, JX663287, JX663329, JX663373, JX663416, JX663446, JX663484, JX663521, JX663590, JX663638, JX663685, JX663727, JX663770, JX663809, JX663847, JX663880, JX663923, JX663970, JX664012, JX664056, JX664097, JX664133, JX664171, JX664215, JX664260, JX664305, JX664347, JX664392, JX664428, JX664464, JX664501, JX664542, JX664586, JX664629, JX664665, JX664697, JX664734, JX664779, JX664818, JX664859, JX664902, JX664947, JX665012

Licania heteromorpha Benth. - Chrysobalanaceae - NC_024062

Linum usitatissimum L. - Linaceae - JX661787, JX661823, JX661866, JX661911, JX661951, JX661987, JX662029, JX662072, JX662159, JX662203, JX662245, JX662286, JX662327, JX662367, JX662406, JX662441, JX662474, JX662509, JX662550, JX662587, JX662629, JX662674, JX662718, JX662760, JX662808, JX662848, JX662921, JX662962, JX663002, JX663040, JX663077, JX663111, JX663158, JX663203, JX663245, JX663288, JX663330, JX663374, JX663417, JX663447, JX663485, JX663522, JX663551, JX663591, JX663639, JX663686, JX663728, JX663771, JX663810, JX663848, JX663881, JX663924, JX663971, JX664013, JX664057, JX664098, JX664134, JX664172, JX664216, JX664261, JX664306, JX664348, JX664393, JX664429, JX664465, JX664502, JX664543, JX664587, JX664666, JX664735, JX664780, JX664819, JX664860, JX664903, JX664948, JX665013

Mammea americana L. - Calophyllaceae - JX661824, JX661867, JX661912, JX661952, JX661988, JX662030, JX662073, JX662117, JX662160, JX662204, JX662246, JX662287, JX662328, JX662368, JX662407, JX662442, JX662475, JX662510, JX662551, JX662588, JX662630, JX662675, JX662719, JX662761, JX662809, JX662849, JX662886, JX662922, JX662963, JX663003, JX663041, JX663078, JX663112, JX663159, JX663204, JX663246, JX663289, JX663331, JX663375, JX663418, JX663448, JX663486, JX663523, JX663592, JX663640, JX663687, JX663729, JX663772, JX663811, JX663849, JX663882, JX663925, JX663972, JX664014, JX664058, JX664099, JX664135, JX664173, JX664217, JX664262, JX664307, JX664349, JX664394, JX664430, JX664466, JX664503, JX664544, JX664588, JX664630, JX664667, JX664698, JX664736, JX664781, JX664820, JX664861, JX664904, JX664949, JX664977, JX665014

Medusagyne oppositifolia Baker - Medusagynaceae - JX661788, JX661825, JX661868, JX661913, JX661953, JX661989, JX662031, JX662074, JX662118, JX662161, JX662205, JX662247, JX662288, JX662329, JX662369, JX662511, JX662589, JX662631, JX662676, JX662720, JX662762, JX662810, JX662850, JX662964, JX663042, JX663113, JX663160, JX663205, JX663247, JX663290, JX663332, JX663376, JX663419, JX663449, JX663487, JX663552, JX663593, JX663641, JX663688, JX663730, JX663773, JX663812, JX663926, JX663973, JX664015, JX664059, JX664100, JX664136, JX664174, JX664218, JX664263, JX664308, JX664350, JX664395, JX664467, JX664545, JX664589, JX664631, JX664699, JX664737, JX664782, JX664821, JX664862, JX664905, JX664950, JX664978, JX665015

Microdesmis caseariifolia Planch. ex Hook. f. - Pandaceae - JX661789, JX661826, JX661869, JX661914, JX661954, JX661990, JX662032, JX662075, JX662119, JX662162, JX662206, JX662248, JX662289, JX662330, JX662370, JX662408, JX662443, JX662476, JX662512, JX662552, JX662590, JX662632, JX662677, JX662721, JX662763, JX662811, JX662851, JX662887, JX662923, JX662965, JX663004, JX663043, JX663079, JX663114, JX663161, JX663206, JX663248, JX663291, JX663333, JX663377, JX663420, JX663450, JX663488, JX663524, JX663553, JX663594, JX663642, JX663689, JX663731, JX663774, JX663813, JX663850, JX663883, JX663927, JX663974, JX664016, JX664060, JX664101, JX664137, JX664175, JX664219, JX664264, JX664309, JX664351, JX664396, JX664431, JX664468, JX664504, JX664546, JX664590, JX664632, JX664668, JX664700, JX664738, JX664783, JX664863, JX664906, JX664951, JX664979, JX665016

Ouratea sp. - Ochnaceae - JX661827, JX661870, JX661915, JX661955, JX661991, JX662033, JX662076, JX662120, JX662163, JX662207, JX662249, JX662290, JX662331, JX662371, JX662409, JX662444, JX662477, JX662513, JX662553, JX662591, JX662633, JX662678, JX662722, JX662764, JX662812, JX662852, JX662888, JX662924, JX662966, JX663044, JX663080, JX663115, JX663162, JX663207, JX663249, JX663292, JX663334, JX663378, JX663421, JX663489, JX663525, JX663595, JX663643, JX663690, JX663732, JX663775, JX663814, JX663851, JX663884, JX663928, JX663975, JX664017, JX664061, JX664102, JX664138, JX664176, JX664220, JX664265, JX664310, JX664352, JX664397, JX664432,

JX664469, JX664505, JX664547, JX664591, JX664633, JX664669, JX664701, JX664739, JX664784, JX664822, JX664864, JX664907, JX664952, JX664980, JX665017

Passiflora ciliata Aiton - Passifloraceae - JX661790, JX661828, JX661871, JX661916, JX661956, JX661992, JX662034, JX662077, JX662164, JX662208, JX662250, JX662291, JX662332, JX662372, JX662410, JX662445, JX662478, JX662514, JX662554, JX662592, JX662634, JX662679, JX662723, JX662765, JX662813, JX662889, JX662925, JX662967, JX663045, JX663081, JX663116, JX663163, JX663208, JX663250, JX663293, JX663335, JX663379, JX663422, JX663451, JX663490, JX663526, JX663554, JX663596, JX663644, JX663691, JX663733, JX663776, JX663815, JX663852, JX663885, JX663929, JX663976, JX664018, JX664062, JX664103, JX664139, JX664177, JX664221, JX664266, JX664311, JX664353, JX664398, JX664433, JX664470, JX664506, JX664548, JX664592, JX664634, JX664670, JX664740, JX664785, JX664823, JX664865, JX664908, JX664953, JX665018

Pera bumeliifolia Griseb. - Peraceae - JX661791, JX661829, JX661872, JX661917, JX661957, JX661993, JX662035, JX662078, JX662121, JX662165, JX662209, JX662251, JX662292, JX662333, JX662373, JX662411, JX662446, JX662479, JX662515, JX662555, JX662593, JX662635, JX662680, JX662724, JX662766, JX662814, JX662853, JX662890, JX662926, JX662968, JX663005, JX663046, JX663082, JX663117, JX663164, JX663209, JX663251, JX663294, JX663336, JX663380, JX663423, JX663452, JX663491, JX663527, JX663555, JX663597, JX663645, JX663692, JX663734, JX663777, JX663816, JX663853, JX663886, JX663930, JX663977, JX664019, JX664063, JX664104, JX664140, JX664178, JX664222, JX664267, JX664312, JX664354, JX664399, JX664434, JX664471, JX664507, JX664549, JX664593, JX664635, JX664671, JX664702, JX664741, JX664786, JX664824, JX664866, JX664909, JX664954, JX664981, JX665019

Ploiarium sp. - Bonnetiaceae - JX661792, JX661831, JX661874, JX661919, JX661959, JX661995, JX662037, JX662080, JX662123, JX662167, JX662211, JX662253, JX662294, JX662335, JX662375, JX662413, JX662448, JX662481, JX662517, JX662557, JX662595, JX662637, JX662726, JX662768, JX662816, JX662855, JX662892, JX662928, JX662970, JX663007, JX663048, JX663084, JX663119, JX663166, JX663211, JX663253, JX663296, JX663338, JX663382, JX663425, JX663454, JX663493, JX663529, JX663556, JX663599, JX663647, JX663694, JX663736, JX663779, JX663818, JX663855, JX663888, JX663932, JX663979, JX664021, JX664065, JX664106, JX664142, JX664179, JX664224, JX664269, JX664314, JX664356, JX664401, JX664436, JX664473, JX664509, JX664551, JX664595, JX664637, JX664673, JX664703, JX664743, JX664788, JX664826, JX664868, JX664911, JX664956, JX664983, JX665021

Podocalyx loranthoides Klotzsch - Picrodendraceae - JX661793, JX661832, JX661875, JX661960, JX661996, JX662038, JX662081, JX662124, JX662168, JX662212, JX662295, JX662376, JX662414, JX662518, JX662638, JX662682, JX662727, JX662769, JX662817, JX662856, JX662929, JX662971, JX663049, JX663120, JX663167, JX663212, JX663297, JX663339, JX663383, JX663426, JX663494, JX663557, JX663600, JX663648, JX663695, JX663737, JX663780, JX663819, JX663933, JX663980, JX664066, JX664180, JX664225, JX664270, JX664315, JX664357, JX664402, JX664437, JX664474, JX664552, JX664596, JX664638, JX664744, JX664789, JX664869, JX664912, JX664957, JX665022

Podostemum ceratophyllum Michx. - Podostemaceae - JX661794, JX661833, JX661876, JX661920, JX661961, JX661997, JX662039, JX662082, JX662169, JX662213, JX662254, JX662296, JX662336, JX662377, JX662415, JX662449, JX662482, JX662519, JX662558, JX662596, JX662639, JX662683, JX662728, JX662770, JX662818, JX662857, JX662893, JX662930, JX662972, JX663008, JX663050, JX663085, JX663121, JX663168, JX663213, JX663254, JX663298, JX663340, JX663384, JX663427, JX663455, JX663495, JX663530,

JX663558, JX663601, JX663649, JX663696, JX663738, JX663781, JX663820, JX663856, JX663889, JX663934, JX663981, JX664022, JX664067, JX664107, JX664143, JX664181, JX664226, JX664271, JX664316, JX664358, JX664403, JX664438, JX664475, JX664510, JX664553, JX664597, JX664639, JX664674, JX664745, JX664790, JX664827, JX664870, JX664913, JX664958, JX665023

Populus deltoides W.Bartram ex Marshall - Salicaceae - MK267316

Putranjiva roxburghii Wall. - Putranjivaceae - JX661877, JX661921, JX661998, JX662040, JX662083, JX662170, JX662214, JX662255, JX662297, JX662337, JX662378, JX662416, JX662450, JX662520, JX662559, JX662597, JX662640, JX662684, JX662729, JX662771, JX662819, JX662858, JX662894, JX662931, JX662973, JX663009, JX663051, JX663122, JX663169, JX663214, JX663255, JX663341, JX663385, JX663428, JX663456, JX663496, JX663602, JX663650, JX663739, JX663782, JX663857, JX663890, JX663935, JX663982, JX664023, JX664068, JX664108, JX664144, JX664182, JX664227, JX664272, JX664317, JX664359, JX664439, JX664476, JX664511, JX664554, JX664598, JX664640, JX664675, JX664746, JX664791, JX664828, JX664871, JX664959, JX665024

Quiina glaziovii Engl. - Quiinaceae - JX661795, JX661834, JX661878, JX661999, JX662041, JX662084, JX662125, JX662171, JX662215, JX662298, JX662560, JX662641, JX662685, JX662772, JX662820, JX662895, JX662974, JX663010, JX663052, JX663123, JX663170, JX663215, JX663299, JX663342, JX663386, JX663429, JX663497, JX663559, JX663603, JX663651, JX663697, JX663740, JX663821, JX663936, JX663983, JX664069, JX664228, JX664273, JX664318, JX664360, JX664404, JX664555, JX664599, JX664641, JX664704, JX664747, JX664792, JX664914, JX664960, JX664984, JX665025

Rhizophora mangle L. - Rhizophoraceae - JX661796, JX661835, JX661879, JX661922, JX661962, JX662000, JX662042, JX662085, JX662126, JX662172, JX662216, JX662256, JX662299, JX662338, JX662379, JX662417, JX662451, JX662483, JX662521, JX662561, JX662598, JX662642, JX662686, JX662730, JX662773, JX662821, JX662859, JX662896, JX662932, JX662975, JX663053, JX663086, JX663124, JX663171, JX663216, JX663256, JX663300, JX663343, JX663387, JX663430, JX663457, JX663498, JX663531, JX663560, JX663604, JX663652, JX663698, JX663741, JX663783, JX663822, JX663858, JX663891, JX663937, JX663984, JX664024, JX664070, JX664109, JX664145, JX664183, JX664229, JX664274, JX664319, JX664361, JX664405, JX664440, JX664477, JX664512, JX664556, JX664600, JX664642, JX664676, JX664705, JX664748, JX664793, JX664829, JX664872, JX664915, JX664961, JX665026

Schistostemon retusum (Ducke) Cuatrec. - Humiriaceae - JX661797, JX661880, JX661923, JX661963, JX662001, JX662043, JX662086, JX662127, JX662173, JX662217, JX662257, JX662300, JX662380, JX662522, JX662562, JX662643, JX662687, JX662731, JX662774, JX662822, JX662860, JX662897, JX662976, JX663011, JX663054, JX663125, JX663172, JX663217, JX663257, JX663301, JX663344, JX663388, JX663431, JX663499, JX663561, JX663605, JX663653, JX663699, JX663742, JX663784, JX663823, JX663938, JX663985, JX664025, JX664071, JX664184, JX664230, JX664275, JX664320, JX664362, JX664406, JX664478, JX664557, JX664601, JX664643, JX664706, JX664749, JX664794, JX664873, JX664916, JX664962, JX665027

Scyphostegia borneensis Stapf. - Salicaceae - JX661798, JX661836, JX661881, JX662044, JX662128, JX662174, JX662218, JX662258, JX662339, JX662381, JX662418, JX662523, JX662563, JX662599, JX662644, JX662688, JX662732, JX662775, JX662823, JX662861, JX662898, JX662977, JX663012, JX663055, JX663173, JX663218, JX663258, JX663345, JX663389, JX663500, JX663606, JX663654, JX663700, JX663743, JX663824, JX663892,

JX663939, JX663986, JX664026, JX664072, JX664110, JX664146, JX664185, JX664231, JX664276, JX664321, JX664407, JX664441, JX664479, JX664558, JX664602, JX664750, JX664795, JX664830, JX664874, JX664917, JX664963, JX665028

Trigonia sp. - Trigoniaceae - JX661882, JX661964, JX662045, JX662087, JX662129, JX662219, JX662259, JX662301, JX662340, JX662524, JX662564, JX662600, JX662645, JX662689, JX662776, JX662899, JX662978, JX663013, JX663056, JX663126, JX663174, JX663219, JX663259, JX663302, JX663346, JX663390, JX663432, JX663458, JX663501, JX663607, JX663701, JX663744, JX663825, JX663940, JX663987, JX664027, JX664073, JX664111, JX664186, JX664232, JX664277, JX664322, JX664363, JX664408, JX664603, JX664644, JX664751, JX664796, JX664831, JX664918, JX664964, JX665029

Turnera ulmifolia L. - Passifloraceae - JX661799, JX661837, JX661883, JX661924, JX661965, JX662002, JX662046, JX662088, JX662175, JX662220, JX662260, JX662302, JX662341, JX662382, JX662419, JX662452, JX662484, JX662565, JX662601, JX662646, JX662690, JX662733, JX662777, JX662824, JX662933, JX662979, JX663057, JX663127, JX663175, JX663220, JX663260, JX663303, JX663347, JX663391, JX663433, JX663502, JX663532, JX663562, JX663608, JX663655, JX663702, JX663745, JX663785, JX663826, JX663859, JX663893, JX663941, JX663988, JX664028, JX664074, JX664112, JX664147, JX664187, JX664233, JX664278, JX664323, JX664364, JX664442, JX664480, JX664513, JX664559, JX664604, JX664645, JX664677, JX664752, JX664797, JX664832, JX664875, JX664965, JX665030

Viola pubescens Aiton - Violaceae - JX661800, JX661838, JX661884, JX661925, JX661966, JX662003, JX662047, JX662089, JX662130, JX662176, JX662221, JX662261, JX662303, JX662342, JX662383, JX662420, JX662453, JX662485, JX662525, JX662566, JX662602, JX662647, JX662691, JX662734, JX662778, JX662825, JX662862, JX662900, JX662934, JX662980, JX663014, JX663058, JX663087, JX663128, JX663176, JX663221, JX663261, JX663304, JX663348, JX663392, JX663434, JX663503, JX663533, JX663563, JX663609, JX663656, JX663703, JX663746, JX663786, JX663827, JX663860, JX663894, JX663942, JX663989, JX664029, JX664075, JX664113, JX664148, JX664188, JX664234, JX664279, JX664324, JX664365, JX664409, JX664443, JX664481, JX664514, JX664560, JX664605, JX664646, JX664678, JX664753, JX664798, JX664833, JX664876, JX664919, JX664966, JX665031

Vismia ferruginea Kunth - Hypericaceae - JX661839, JX661885, JX661926, JX661967, JX662004, JX662048, JX662090, JX662131, JX662177, JX662222, JX662262, JX662304, JX662343, JX662384, JX662421, JX662454, JX662486, JX662526, JX662567, JX662603, JX662648, JX662692, JX662735, JX662779, JX662826, JX662863, JX662935, JX662981, JX663015, JX663059, JX663129, JX663177, JX663222, JX663262, JX663305, JX663349, JX663393, JX663435, JX663504, JX663534, JX663610, JX663657, JX663704, JX663747, JX663787, JX663828, JX663861, JX663895, JX663943, JX663990, JX664030, JX664076, JX664114, JX664149, JX664189, JX664235, JX664280, JX664325, JX664366, JX664444, JX664482, JX664515, JX664561, JX664606, JX664647, JX664679, JX664754, JX664799, JX664834, JX664877, JX664967, JX665032
